# Restoring O-glycosylation and expression of MUC2 limits progression of colorectal cancer

**DOI:** 10.1038/s41419-026-08981-x

**Published:** 2026-06-21

**Authors:** Yian Yang, Yuesong Yin, Wei Xu, Yan Kang, Jiawei Chen, Yanfeng Zou, Xuan Wang, Zhigang Xiao, Peiguo Cao, Zheng Li

**Affiliations:** 1 Department of Oncology, the Third Xiangya Hospital, Central South University, Changsha, China; 2 Department of Orthopedics, the Third Xiangya Hospital, Central South University, Changsha, China; 3 NHC Key Laboratory of Carcinogenesis, Cancer Research Institute, School of Basic Medical Science, Central South University, Changsha, China; 4 Department of General Surgery, The First Affiliated Hospital of Hunan Normal University (Hunan Provincial People’s Hospital), Changsha, China

**Keywords:** Cancer, Predictive markers

## Abstract

Colorectal cancer (CRC) progression is associated with intestinal barrier dysfunction, yet the molecular mechanisms governing mucin glycosylation remain unclear. This study aims to investigate the regulatory pathway controlling MUC2 expression and O-glycosylation in CRC and assess the therapeutic potential of rosiglitazone in restoring mucosal integrity. In this study, B3GNT6 was identified as a key glycosyltransferase that promotes MUC2 O-glycosylation and protein stability. KLF4 was shown to transcriptionally regulate both B3GNT6 and MUC2, with its activity modulated by PPARg. Treatment with the PPARg agonist rosiglitazone activated the PPARg-KLF4-B3GNT6 axis, restoring MUC2 function and enhancing the mucus barrier integrity. These findings highlight a novel therapeutic avenue for CRC by targeting mucin glycosylation.

**Figure:**
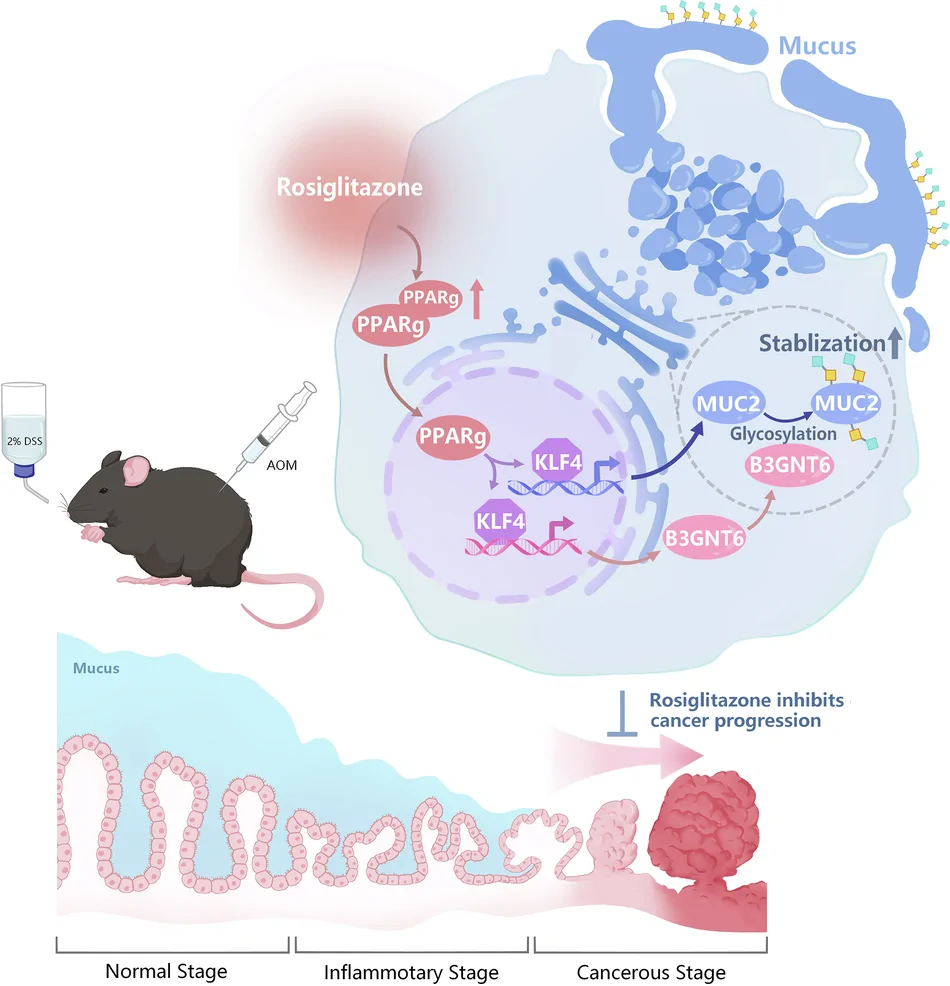

## Introduction

Colorectal cancer (CRC) is the fourth leading cause of cancer-related mortality globally [1]. CRC development is strongly associated with genetic and environmental factors, particularly those impacting the intestinal barrier [2, 3], which plays a pivotal role in preventing disease. Disruption of this barrier can lead to increased permeability, allowing harmful substances and pathogens to enter the bloodstream [4, 5], thereby contributing to inflammation and tumorigenesis. Emerging research highlights the significance of the gut microbiota and its interaction with the mucosal layer in influencing CRC development.

MUC2 is a member of the mucin family, contributing to intestinal mucus gel formation through glycosylation, thus creating a protective layer that protects the intestinal epithelium and maintains the mucosal barrier [6, 7]. MUC2 function is shaped by glycosylation, a vital post-translational modification [8–10]. Highly glycosylated modifications ensure that MUC2 can form gels when combined with substantial quantities of water, while aberrant glycosylation disrupts normal mucus structure [11, 12]. Dysregulated MUC2 expression and aberrant glycosylation have been implicated in increased intestinal permeability and compromised barrier function [13, 14]. However, the specific mechanisms underlying these alterations in CRC remain poorly understood.

In this study, we identify B3GNT6 as a key glycosyltransferase that protects MUC2 from bacterial degradation via O-glycosylation, and demonstrate that rosiglitazone-activated PPARg-KLF4-B3GNT6 axis restores MUC2 glycosylation and barrier integrity, thereby suppressing CRC progression in vitro and in vivo. These findings uncover a previously unrecognized mechanism linking transcriptional regulation, mucin glycosylation, and barrier function in CRC, and suggest that targeting this PPARg-mediated protective pathway represents a potential therapeutic strategy for maintaining mucosal homeostasis and combating CRC development.

## Results

### Downregulation of MUC2 expression and O-glycosylation in colorectal cancer

**Fig. 1:**
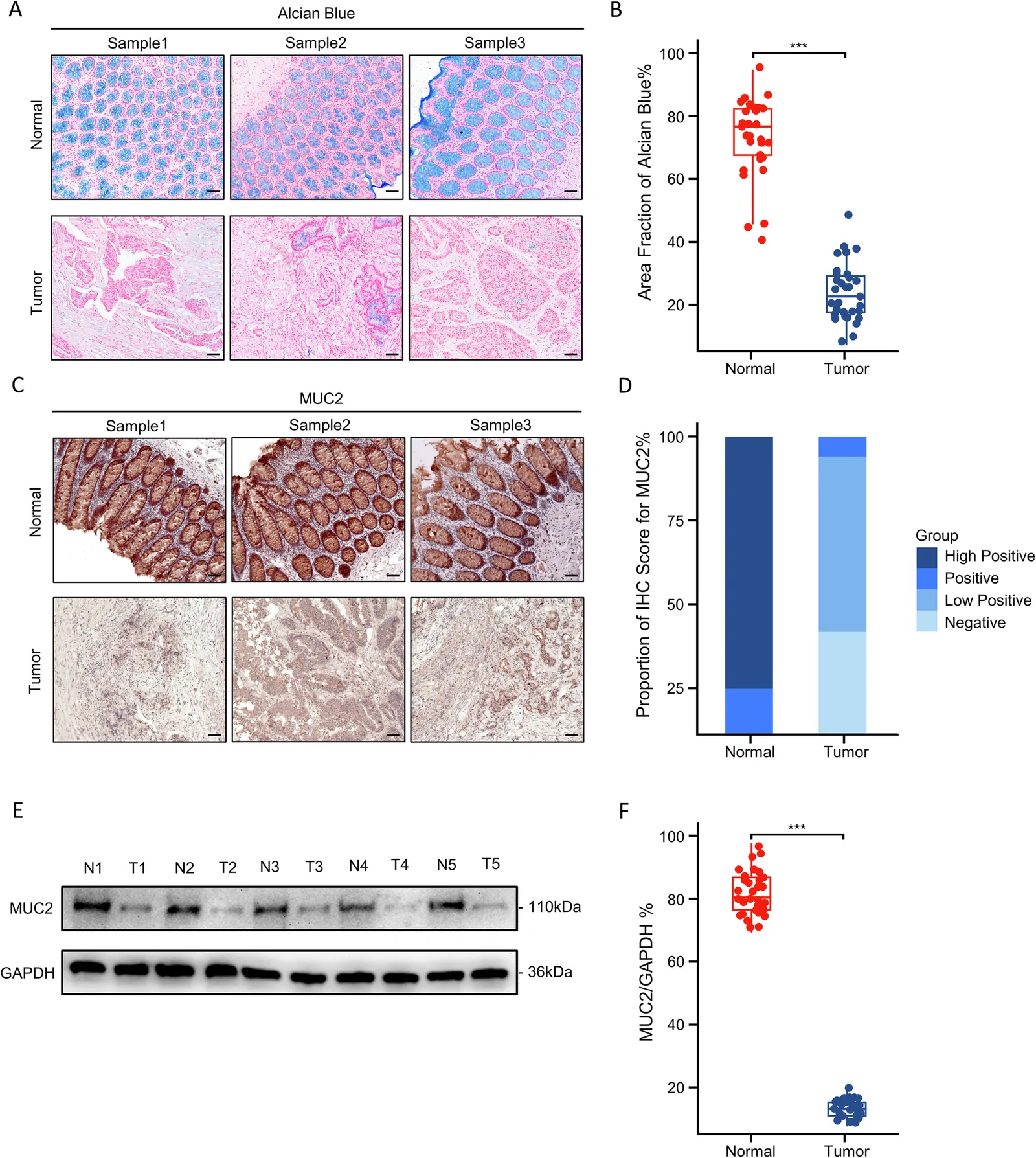
Reduced MUC2 expression in colorectal cancer tissues. **A**, **B** Detection and quantification of intestinal mucus by Alcian Blue staining in paired tissues (*n* = 31, normal; *n* = 31, tumor). Scale bars, 50 µm. **C**, **D** IHC staining and quantification of MUC2 in CRC tissues (*n* = 31, normal; *n* = 31, tumor). Scale bars, 50 µm. **E**, **F** Western blot of MUC2 and corresponding densitometry quantification (*n* = 31, normal; *n* = 31, tumor). Data were analyzed by paired t-test. Error bars represent mean ± SEM. *p < 0.05, **p < 0.01, ***p < 0.001. IHC immunohistochemistry, CRC colorectal cancer.

While investigating the clinical relevance and functional role of MUC2 in colorectal cancer (CRC), MUC2 was found to be highly expressed in normal gastrointestinal tissues based on GTEx data (Fig. S1A) but significantly downregulated in CRC tumors compared to adjacent normal tissues in the TCGA cohort (Fig. S1B). IHC staining demonstrated reduced MUC2 protein expression in CRC tissues (Fig. S1C). Survival analysis suggested a non-significant trend toward poorer overall survival in the low MUC2 expression group (Fig. S1D). To validate these findings, Alcian blue staining (Fig. 1A, B) was performed to assess intestinal mucus production. IHC staining (Fig. 1C, D) and Western blots performed on patient samples confirmed reduced mucus production and lower MUC2 protein levels in tumors (Fig. 1E, F), suggesting a compromised intestinal barrier.

Analysis of cell lines showed similarly decreased MUC2 expression in CRC cells compared to normal colonic epithelial cells (Fig. S1E). To explore its potential functional role, MUC2 expression was silenced using siRNA, which led to increased colony formation and cell proliferation (Fig. S1F–H).

These results suggest that MUC2 downregulation may contribute to CRC progression by promoting tumor cell growth.

### B3GNT6 facilitates MUC2 core3 O-Glycosylation and enhances stability

To identify genes associated with MUC2 regulation in colorectal cancer, Pearson correlation analysis was performed using TCGA data. B3GNT6 showed the strongest positive correlation with MUC2 expression (Fig. S2A, B) and was significantly downregulated in tumors based on boxplot and correlation analysis (Fig. S2C, D).

GO enrichment showed significant involvement of MUC2-associated genes in O-glycosylation (Fig. S2E), while KEGG analysis highlighted enrichment in mucin type O-glycan biosynthesis (Fig. S2F). Although aberrant O-glycosylation has been implicated in colorectal carcinogenesis and reduced MUC2 [15], the underlying regulatory mechanisms remain unclear.

**Fig. 2:**
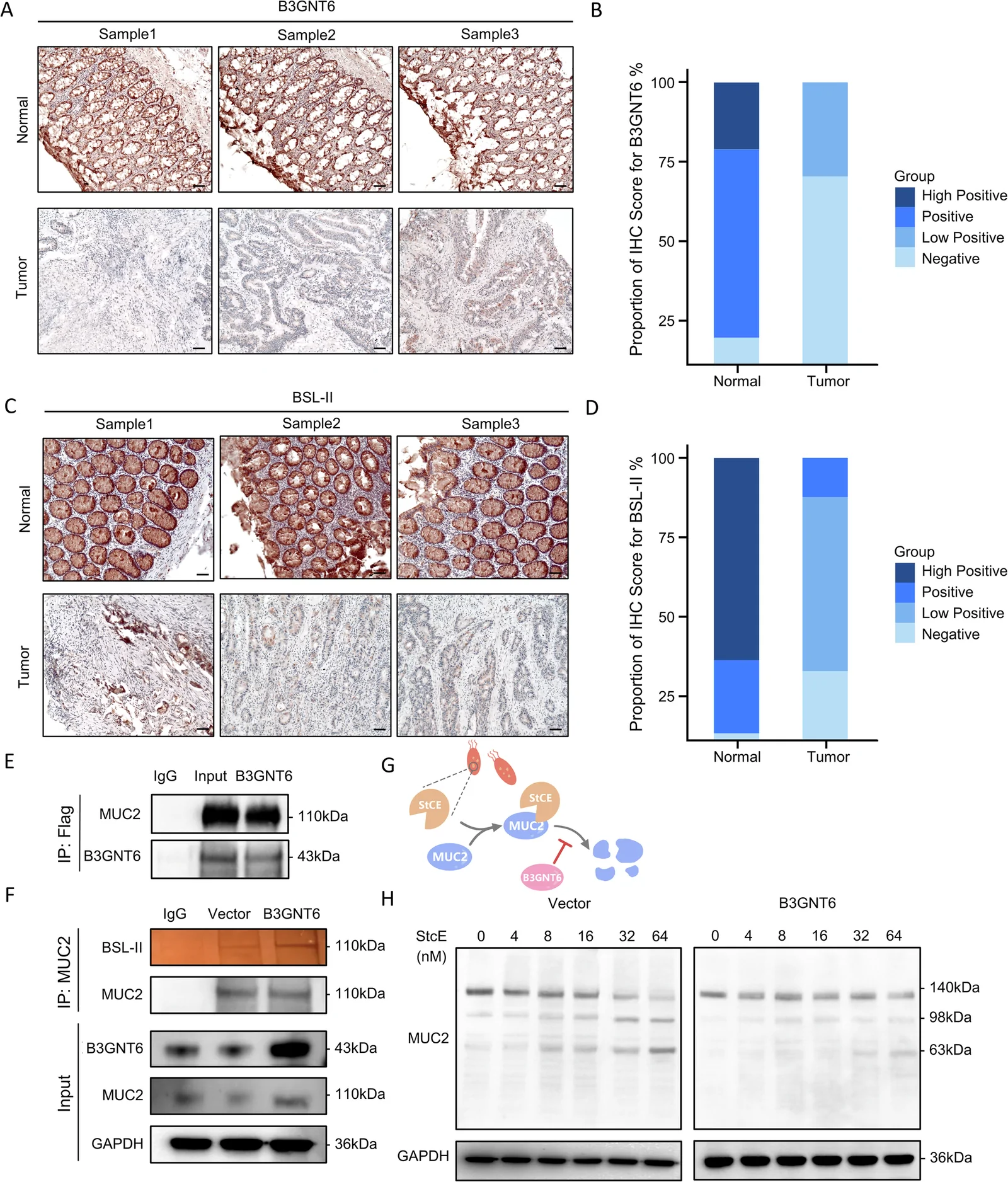
B3GNT6 enhances MUC2 protein expression and stability. **A**, **B** IHC staining and quantification of B3GNT6 in CRC tissues. Scale bars, 50 µm. **C**, **D** BSL-II lectin staining and quantification of Core3 O-glycan in CRC tissues. **E** Co-IP analysis of MUC2-B3GNT6 interaction after transfection with pcDNA3.1-B3GNT6-Flag antibody (*n* = 3, Input; *n* = 3, IgG; *n* = 3, B3GNT6). **F** Co-IP and lectin analysis showing Core3 glycosylation of MUC2 pulled down by anti-MUC2 antibody. **G**, **H** StcE-mediated cleavage of MUC2 and Western blot showing degradation after treatment with increasing concentrations (0–64 nM) for 3 h. Data were analyzed by one-way ANOVA with Tukey’s post-hoc test. Error bars represent mean ± SEM. *p < 0.05, **p < 0.01, ***p < 0.001. Co-IP co-immunoprecipitation, IHC immunohistochemistry, CRC colorectal cancer, PPI protein-protein interaction, GSEA gene set enrichment analysis, IHC immunohistochemistry, Co-IP co-immunoprecipitation, StcE Recombinant Escherichia coli O157: H7 Metalloprotease.

To further explore the relationship between MUC2 and glycosylation enzymes, GSEA and PPI network analysis were performed. B3GNT6 was identified as the top-ranking glycosyltransferase associated with MUC2 expression (Fig. S2G, H). IHC staining on 31 paired CRC and adjacent normal tissues confirmed significantly reduced or absent B3GNT6 expression in tumors (Fig. 2A, B). Since B3GNT6 catalyzes the synthesis of the Core 3 O-glycan structure [16], a key modification in mucin-type glycoproteins (Fig. S3A), it was hypothesized that B3GNT6 modulates MUC2 O-glycosylation and affects its expression in colorectal cancer.

To assess this hypothesis, the effect of B3GNT6 overexpression on MUC2 expression and glycosylation was examined in vitro. B3GNT6 overexpression led to increased MUC2 mRNA and protein levels (Fig. S3B, C). Lectin staining with BSL-II, which binds specifically to Core 3 O-glycans [17], showed a reduction in Core 3 O-glycosylation in CRC tissues compared to adjacent normal tissues (Fig. 2C, D). In contrast, BSL-II staining was significantly enhanced in B3GNT6-overexpressing LOVO cells, indicating increased Core 3 modification (Fig. S3D). Immunoprecipitation further revealed a direct interaction between B3GNT6 and MUC2 (Fig. 2E), and BSL-II staining confirmed that B3GNT6 overexpression enhanced Core 3 O-glycosylation on MUC2 (Fig. 2F).

To assess whether B3GNT6-mediated glycosylation contributes to MUC2 stability, StcE, a metalloprotease secreted by EHEC [18] that cleaves O-glycosylated peptides and impairs mucin barrier function [19, 20], was utilized. Overexpression of B3GNT6 reduced the levels of MUC2 fragments produced by StcE treatment in LOVO cells (Fig. 2G, H), while ELISA detected elevated MUC2 levels in the culture supernatant (Fig. S3E, F). These findings suggest that B3GNT6 promotes Core 3 O-glycosylation of MUC2 and may enhance its stability, potentially contributing to restoration of the intestinal mucus barrier in CRC.

### B3GNT6 restricts CRC cell growth in a MUC2-dependent manner in vitro and in vivo

Given that MUC2 and its O-glycosylation are known to protect against colitis [21] and colorectal cancer, we performed rescue experiments in CRC cells to test whether B3GNT6 inhibits cell proliferation through MUC2. Consistent with our hypothesis, B3GNT6 overexpression significantly reduced cell proliferation in both LOVO and HT29 cells, while B3GNT6 knockdown promoted cell growth. In contrast, no significant changes in migration or invasion were detected following B3GNT6 manipulation (Fig. S4A–F). Furthermore, knockdown of MUC2 markedly attenuated the inhibitory effect of B3GNT6 overexpression, leading to a significant increase in EdU-positive cells and restoration of cell proliferation (Fig. S5A–F). These findings indicate that the anti-proliferative effect of B3GNT6 is largely dependent on MUC2, supporting a model in which B3GNT6 acts upstream of MUC2 to regulate CRC cell proliferation.

**Fig. 3:**
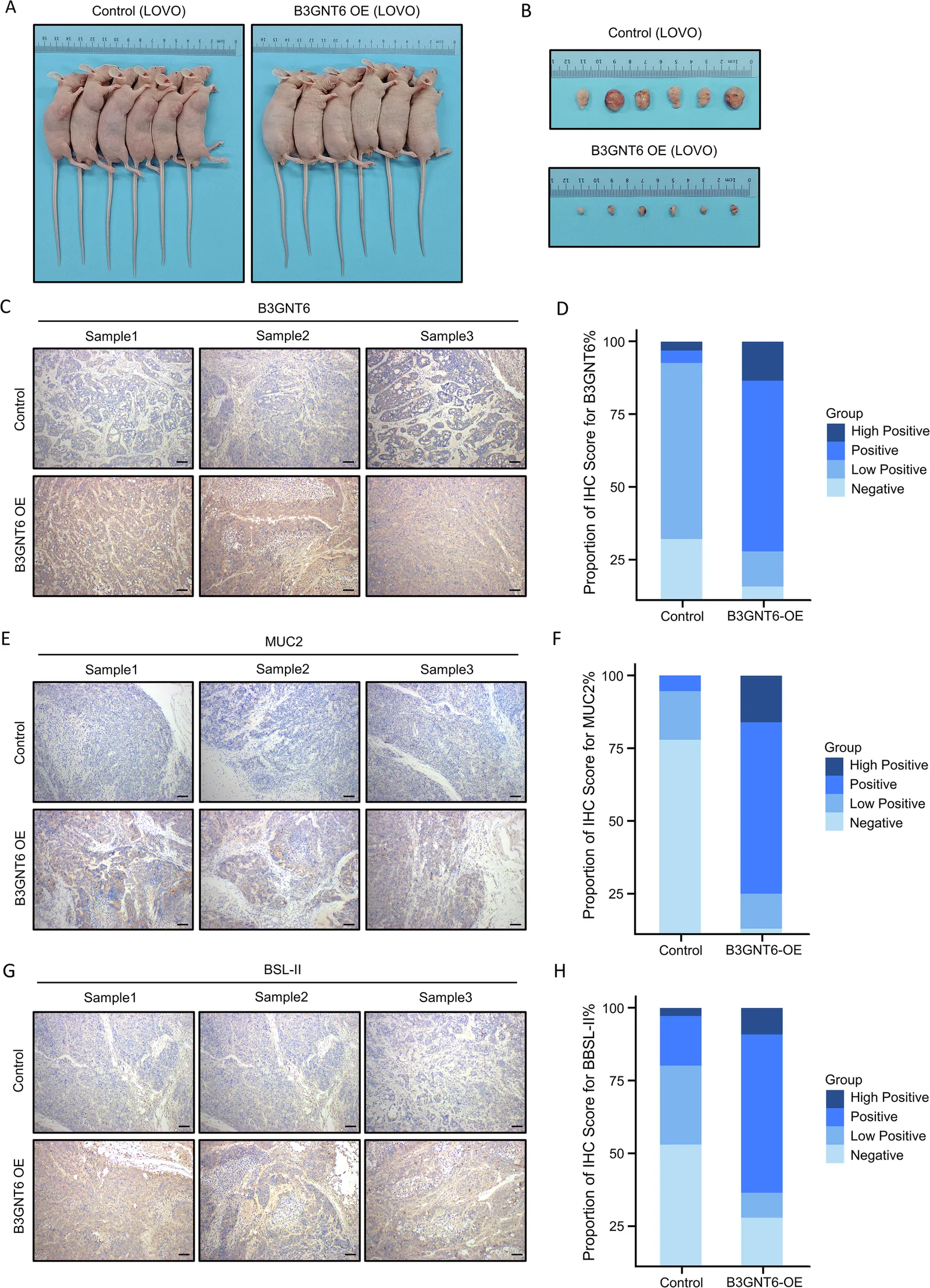
B3GNT6 overexpression suppresses CRC xenograft growth and enhances MUC2 expression and mucin O-glycosylation in vivo. **A** Representative images of nude mice bearing subcutaneous xenograft tumors derived from control and B3GNT6 overexpression (B3GNT6 OE) LOVO cells. **B** Representative images of excised tumors from each group at the endpoint. **C** Representative immunohistochemical staining of B3GNT6 in xenograft tumor tissues from the control and B3 OE groups. **D** Quantification of B3GNT6 immunohistochemical staining scores. **E** Representative immunohistochemical staining of MUC2 in xenograft tumor tissues from the control and B3 OE groups. **F** Quantification of MUC2 immunohistochemical staining scores. **G** Representative BSL-II lectin staining of xenograft tumor tissues from the control and B3 OE groups. **H** Quantification of BSL-II staining scores. Data are presented as mean ± SEM. Statistical significance was determined using an unpaired two-tailed Student’s t-test. *P < 0.05, **P < 0.01, ***P < 0.001.

To further establish a functional link between B3GNT6 and colorectal cancer progression in vivo, we generated subcutaneous xenografts using control and B3GNT6-overexpressing LOVO cells in nude mice. Compared with the control group, tumors derived from B3GNT6-overexpressing cells were markedly smaller at the experimental endpoint, as shown by representative images of tumor-bearing mice and excised xenografts (Fig. 3A, B). These findings indicate that B3GNT6 overexpression suppresses the tumorigenic potential of CRC cells in vivo.

To investigate whether the antitumor effect of B3GNT6 was associated with restoration of the MUC2-related molecular phenotype, we next examined B3GNT6, MUC2, and mucin O-glycosylation levels in xenograft tissues. Immunohistochemical staining confirmed increased B3GNT6 expression in tumors from the B3GNT6 OE group compared with controls (Fig. 3C, D). Notably, MUC2 staining was also significantly enhanced in the B3GNT6 OE group (Fig. 3E, F), supporting the notion that B3GNT6 positively regulates MUC2 expression in vivo. In addition, BSL-II lectin staining, which reflects mucin-associated glycosylation status, was markedly increased in the B3GNT6 OE group (Fig. 3G, H), indicating enhanced O-glycosylation in xenograft tumors following B3GNT6 overexpression. Taken together, these results demonstrate that B3GNT6 overexpression inhibits CRC xenograft growth and is accompanied by increased MUC2 expression and elevated mucin glycosylation in vivo, supporting a tumor-suppressive role of the B3GNT6-MUC2 axis in colorectal cancer progression.

### KLF4 functions as a dual transcriptional activator of MUC2 and B3GNT6

To investigate the transcriptional mechanism of B3GNT6-MUC2 axis downregulation in CRC, we analyzed potential transcription factor binding sites in the MUC2 and B3GNT6 promoters using the JASPAR database (http://jaspar.genereg.net). KLF4 was identified as a candidate regulator and showed reduced expression in tumors and a positive correlation with MUC2 and B3GNT6 in TCGA (Fig. S6A–C). Schematic diagrams further illustrated the predicted KLF4 binding sites within the promoter regions (Fig. S6D, E), including a site at −87 to −76 bp upstream of the MUC2 transcription start site, and two sites at −1934 to −1924 bp and −165 to −155 bp within the B3GNT6 promoter.

**Fig. 4:**
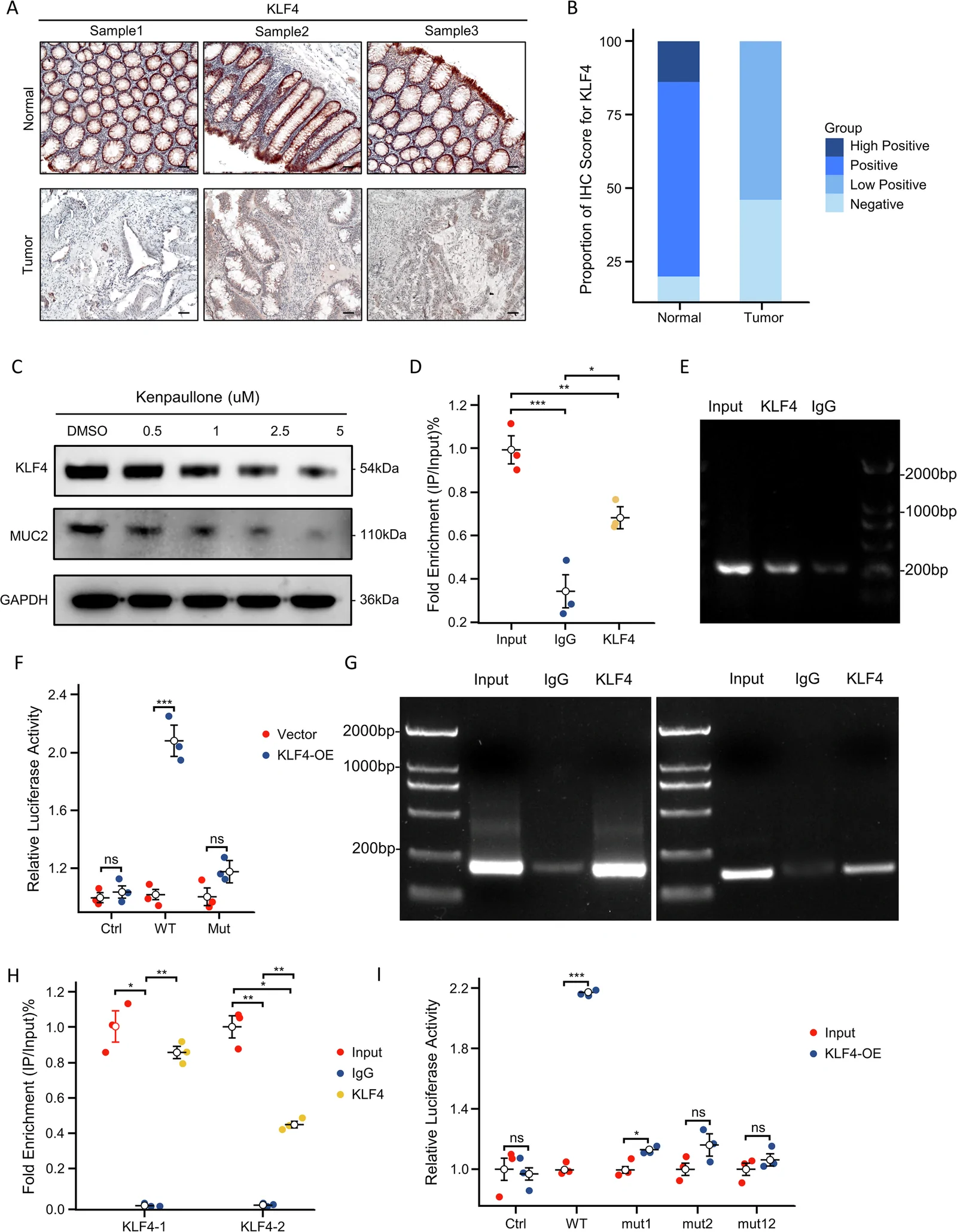
KLF4 directly activates MUC2 and B3GNT6 transcription. **A**, **B** IHC staining and quantification of KLF4 in CRC tissues (*n* = 31, normal; *n* = 31, tumor). Scale bars, 50 µm. **C** Western blot detecting MUC2 after Kenpaullone (KLF4 inhibitor) treatment at indicated doses for 24 h. **D**, **E** ChIP-qPCR showing KLF4 binding to MUC2 promoter site (*n* = 3, Input; *n* = 3, IgG; *n* = 3, CHIP-KLF4). **F** Luciferase reporter assay in LOVO cells co-transfected with wild-type or mutant MUC2 promoter constructs (*n* = 5, Ctrl; *n* = 5, WT; *n* = 5, mut). **G**, **H** ChIP-qPCR confirming KLF4 binding to B3GNT6 promoter (*n* = 3, Input; *n* = 3, IgG; *n* = 3, CHIP-KLF4). **I** Luciferase assay with WT and mutant B3GNT6 promoter constructs (*n* = 5, Ctrl; *n* = 5, WT; *n* = 5, mut1; *n* = 5, mut2; *n* = 5, mut12). Data were analyzed by one-way ANOVA with Tukey’s post-hoc test. Error bars represent mean ± SEM. *p < 0.05, **p < 0.01, ***p < 0.001. ChIP chromatin immunoprecipitation, qPCR quantitative PCR, CRC colorectal cancer.

IHC staining revealed lower KLF4 expression in CRC tissues compared to adjacent normal tissues (Fig. 4A, B). In LOVO cells, inhibition of KLF4 decreased MUC2 levels, while overexpression increased its levels, as shown by qPCR and Western blot (Fig. S6F–I). Western blot analysis also demonstrated a dose-dependent reduction of MUC2 with increasing concentrations of Kenpaullone (Fig. 4C).

Consistently, to confirm direct binding of KLF4 to the MUC2 promoter, ChIP-qPCR validated (Fig. 4D, E) a binding site at 76–87 bp upstream of MUC2’s ATG initiation. Luciferase reporter assays using wild-type and mutant promoter constructs further confirmed that mutation of the KLF4 binding site abolished promoter activity (Fig. S6J, Fig. 4F). In addition, overexpression of KLF4 increased B3GNT6 expression, while siRNA-mediated knockdown reduced its levels (Fig. S6K, L). ChIP-qPCR confirmed two KLF4 binding sites within the B3GNT6 promoter, both of which were associated with reduced luciferase activity when mutated (Fig. S6M, Fig. 4G–I).

These results suggest that KLF4 functions as a transcriptional activator of both MUC2 and B3GNT6, thereby providing a potential strategy to restore MUC2 expression and its O-glycosylation for inhibiting CRC progression.

### PPARg upregulates MUC2 and B3GNT6 via KLF4 activation

**Fig. 5:**
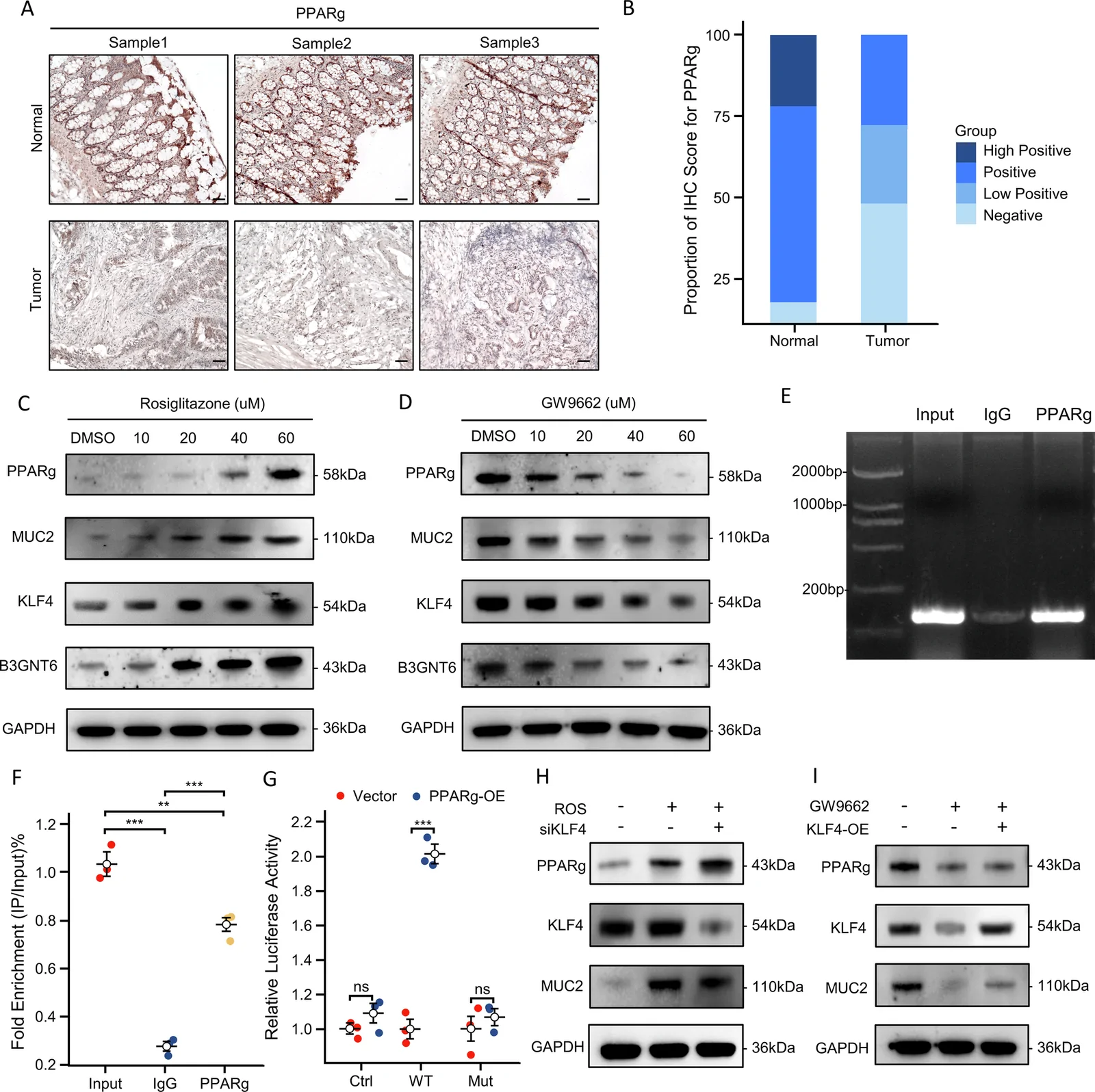
PPARg regulates MUC2 and B3GNT6 expression via KLF4. **A**, **B** IHC staining and quantification of PPARg in CRC tissues (*n* = 31, normal; *n* = 31, tumor). Scale bars, 50 µm. **C**, **D** Western blot of PPARg, KLF4, MUC2, and B3GNT6 after Rosiglitazone or GW9662 treatment at indicated doses. **E**, **F** ChIP-qPCR validating KLF4 binding after PPARg activation (*n* = 3, Input; *n* = 3, IgG; *n* = 3, CHIP-PPARg). **G** Luciferase assay showing KLF4 promoter activity upon PPARg modulation (*n* = 5, Ctrl; *n* = 5, WT; *n* = 5, mut). **H**, **I** Western blot of KLF4/MUC2 in LOVO cells with siKLF4 + Rosiglitazone(40 uM) or KLF4 overexpression + GW9662 (20 uM). Data were analyzed by one-way ANOVA with Tukey’s post-hoc test. Error bars represent mean ± SEM. *p < 0.05, **p < 0.01, ***p < 0.001. ChIP chromatin immunoprecipitation, IHC immunohistochemistry, CRC colorectal cancer.

Although no compounds are currently known to directly target MUC2, B3GNT6, or KLF4, to explore potential pharmacological strategies to modulate this axis, we next investigated transcriptional regulators upstream of KLF4. Peroxisome proliferator-activated receptor γ (PPARg), a nuclear receptor implicated in metabolic regulation and tumor biology, has been reported to exert anti-tumor effects in colorectal cancer by modulating immune evasion and epithelial-mesenchymal transition (EMT) programs [22–24]. Previous studies demonstrated that PPARg can transcriptionally regulate KLF4 in pancreatic cancer cells [25, 26]. Analysis of TCGA-COAD showed that PPARg expression was reduced in CRC tumors compared to normal tissues (Fig. S7A). Although the correlation between PPARg and KLF4 expression was weak (R = 0.137), it was statistically significant (*P* < 0.001, Fig. S7B). This potential association prompted us to further investigate the upstream regulatory mechanism. IHC analysis of patient samples confirmed lower PPARg protein levels in tumors compared to adjacent tissues (Fig. 5A, B).

To assess whether PPARg modulates this regulatory axis, LOVO cells were treated with the PPARg agonist rosiglitazone and the antagonist GW9662. Treatment with rosiglitazone increased the expression of KLF4, B3GNT6, and MUC2, while GW9662 suppressed their expression, consistent with changes in PPARg activity (Fig. 5C, D). These results suggest that PPARg may function as an upstream regulator of this transcriptional program.

Although PPARg binding sites were predicted in the MUC2 promoter, ChIP-qPCR did not detect direct binding, suggesting that MUC2 is not a primary transcriptional target of PPARg (Fig. S7C, D). Instead, JASPAR-based motif analysis predicted a PPARg binding site within the KLF4 promoter, suggesting a potential transcriptional link (Fig. S7E). ChIP and qPCR confirmed that PPARg binds to and activates the KLF4 promoter (Fig. 5E, F), as luciferase activity driven by the KLF4 promoter was abolished upon mutation of the PPARg binding site (Fig. 5G, Fig. S7F, G). To determine whether PPARg-mediated MUC2 upregulation is KLF4-dependent, siKLF4 was transfected into rosiglitazone-treated LOVO cells. Silencing KLF4 impaired the rosiglitazone-induced increase in MUC2 expression, while PPARg inhibition by GW9662 reduced MUC2 expression even in the presence of KLF4, supporting an indirect regulatory pathway (Fig. 5H, I).

To validate these findings in vivo, C57BL/6 mice were fed diets supplemented with rosiglitazone or GW9662 for two weeks. ELISA revealed corresponding increases in Core 3 O-glycosylation and MUC2 secretion, consistent with PPARg activation (Fig. S7H). IHC staining showed increased expression of PPARg, KLF4, B3GNT6, and MUC2 in the rosiglitazone-treated group, while Alcian blue staining showed a thicker mucus layer (Fig. S7I, J).

Collectively, these data suggest that PPARg acts as an upstream transcriptional activator of KLF4, which in turn co-activates MUC2 and B3GNT6 expression. This coordinated regulation restores MUC2 expression and its O-glycosylation, thereby offering a potential therapeutic strategy to inhibit CRC cell proliferation.

### Rosiglitazone suppresses tumor growth via PPARg pathway activation

Given that PPARg acts as an upstream transcriptional activator of the KLF4-MUC2/B3GNT6 axis, we investigated whether its activation by rosiglitazone suppresses CRC progression. Treatment with the PPARg antagonist GW9662 promoted CRC cell proliferation, whereas rosiglitazone suppressed it. KLF4 overexpression reversed the pro-proliferative effect of GW9662, while KLF4 knockdown attenuated the anti-proliferative effect of rosiglitazone (Fig. S8A–D), supporting a PPARg-KLF4-dependent mechanism.

To determine whether rosiglitazone acts through the downstream B3GNT6-MUC2 axis, we performed rescue experiments. The anti-proliferative effect of rosiglitazone was markedly attenuated by B3GNT6 knockdown and further compromised by simultaneous knockdown of both B3GNT6 and MUC2, which restored proliferation rates to levels comparable to or even exceeding those of control cells (Fig. S8E–J). Collectively, these results indicate that the anti-proliferative effect of rosiglitazone is largely mediated by the KLF4-B3GNT6/MUC2 axis.

**Fig. 6:**
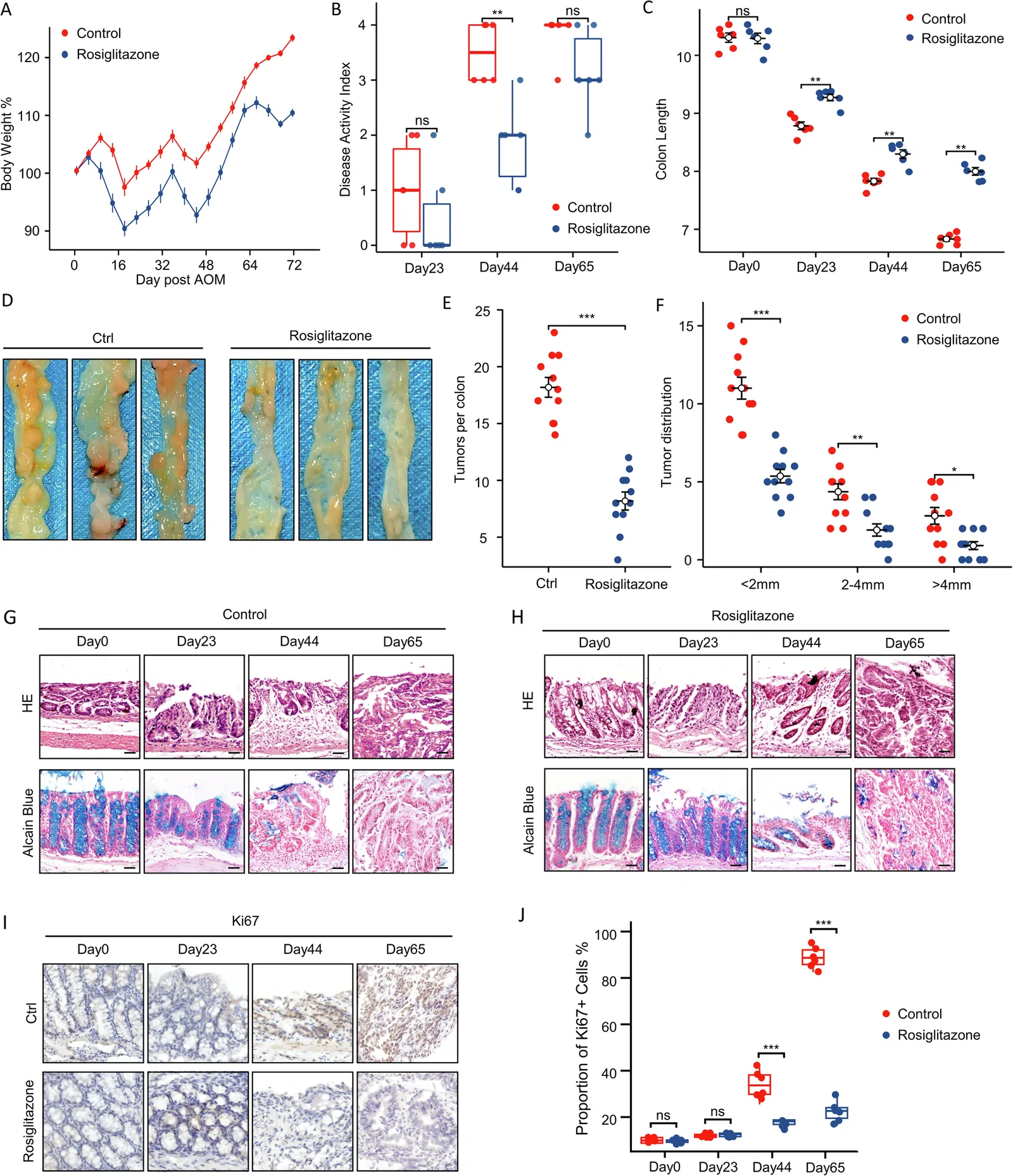
Rosiglitazone reverses the progressive downregulation of PPARg, KLF4, MUC2, and B3GNT6 during colorectal cancer development. **A** Weight change of AOM/DSS mice over time (*n* = 6, AOM/DSS-Control; *n* = 6, AOM/DSS-Rosiglitazone). **B** Disease Activity Index (DAI) based on body weight loss, stool consistency, and bleeding at specified timepoints. **C** Colon length on Days 7, 23, 44, and 65. **D** Representative images of colons at different stages of AOM/DSS treatment. **E** Tumor counts per colon. **F** Tumor size distribution (< 2 mm, 2–4 mm, > 4 mm). **G**, **H** H&E staining, Alcian Blue staining of intestinal mucus in mouse colonic tissues at four stages (Day 0, Day 23, Day 44, Day 65 of DSS cycle; *n* = 6, AOM/DSS-Control; *n* = 6, AOM/DSS-Rosiglitazone). Scale bars, 50 µm. **I**, **J** Staining and quantification of Ki67-positive cells in the same tissues and timepoints (*n* = 6, AOM/DSS-Control; *n* = 6, AOM/DSS-Rosiglitazone). Data were analyzed by one-way ANOVA with Tukey’s post-hoc test. Error bars represent mean ± SEM. *p < 0.05, **p < 0.01, ***p < 0.001. H&E hematoxylin-eosin, DAI Disease Activity Index, CRC colorectal cancer.

To investigate the in vivo effects of rosiglitazone, the AOM/DSS-induced colitis-associated CRC mouse model was utilized. Rosiglitazone-treated mice exhibited significantly lower tumor incidence, tumor burden, and Disease Activity Index (DAI) scores, along with improvements in body weight, colon length, and mucus layer thickness (Fig. 6A–C, Fig. S9A). Macroscopic examination of colonic tissue revealed fewer visible tumors and reduced infiltration in the rosiglitazone group (Fig. 6D). Quantitative analysis confirmed a lower number of tumors across all tumor size categories (Fig. 6E, F). Histological grading of paraffin-embedded colon sections revealed reduced pathological severity in rosiglitazone-treated animals across multiple DSS cycles (Fig. S9B, C, Table S1).

To further assess mucosal integrity, colonic sections were stained with HE and Alcian blue. Rosiglitazone preserved crypt architecture and mucus secretion following the first DSS cycle, indicating a delay in barrier breakdown (Fig. 6G, H). IHC analysis demonstrated maintenance or upregulation of PPARg, KLF4, B3GNT6, MUC2, and Core 3 O-glycosylation in the rosiglitazone group, while these markers were reduced in untreated controls prior to tumor formation (Fig. S9D, E). Ki67 staining further confirmed reduced tumor cell proliferation in rosiglitazone-treated mice (Fig. 6I, J).

In addition, 16S rRNA sequencing showed that in AOM-DSS model, with tumor progression, an increase in mucin-associated bacterial taxa, including Akkermansia muciniphila, Bacteroidales, and Muribaculaceae, was observed. Functional prediction analysis suggested a decline in glycosylation-related pathways during CRC progression. However, rosiglitazone treatment partially altered these microbial compositions, and glycosylation-related pathways appeared to be relatively preserved (Fig. S10).

These findings suggest that rosiglitazone may suppress colorectal tumor development by activating the PPARg-KLF4-B3GNT6/MUC2 axis, thereby maintaining the mucosal barrier and reducing epithelial proliferation.

## Discussion

In this study, we identify a regulatory axis involving PPARg, KLF4, B3GNT6, and MUC2 that collectively maintains intestinal barrier integrity and suppresses colorectal cancer progression. These findings support a conceptual framework in which epithelial O-glycosylation is not merely a structural feature of the mucus layer, but an active and inducible regulatory component of tumor suppression-mechanistically linking barrier function, MUC2 stability, and CRC growth restriction via the PPARg-KLF4-B3GNT6/MUC2 axis. Notably, this tumor-suppressive pathway operates independently of classical differentiation-related mechanisms, highlighting a distinct dimension of glycosylation-mediated mucosal protection.

B3GNT6 is a key enzyme responsible for synthesizing the Core 3 O-glycan structure, which is essential for proper mucin folding and secretion [27, 28]. A growing body of evidence has established that dysregulated mucin-type O-glycosylation is a hallmark of CRC, with Core 1/Core 3 imbalance correlating with tumor adhesion, metastatic dissemination, and adverse clinical outcomes [29]. Our results extend this paradigm by demonstrating that the Core 3 synthase B3GNT6 functions as a critical effector downstream of the PPARg-KLF4 signaling cascade, providing a molecular link between metabolic/nuclear receptor signaling and the glycan-mediated control of epithelial homeostasis.

To assess Core 3 O-glycosylation in clinical and preclinical samples, BSL-II, a lectin specific for Core 3 O-glycans, was employed. BSL-II staining revealed markedly reduced Core 3 modification in colorectal cancer tissues compared to adjacent normal tissues, in both human specimens and the AOM/DSS mouse model. This pattern mirrors observations made with other mucin-recognizing lectins, such as Sambucus nigra agglutinin (SNA) [30] and Agaricus bisporus agglutinin (ABA) [31], which have been proposed as potential biomarkers for early-stage colorectal cancer [32]. These findings suggest that aberrant glycosylation may serve not only as a driver of CRC progression but also as a promising biomarker for early detection and risk stratification.

PPARg has been widely reported to exert anti-tumor effects in CRC, in part through induction of cell cycle arrest and tumor cell death [33, 34] via downstream targets such as KLF4 [35, 36]. In addition to transcriptional regulation, PPARg activation has been reported to induce tumor cell apoptosis via ROS-mediated DNA damage, suggesting a context-dependent anti-tumor mechanism [37]. Low KLF4 expression has been linked to poor survival and high recurrence in CRC patients, consistent with its established role in cell cycle control and tumor suppression [38, 39]. A recent multi-omics study by Saha et al. further mapped PPARg transcriptional networks in colon cancer, identifying 14,000–34,000 PPARG binding sites and 18 hub genes (e.g., PTK2, CDK8, PRKDC) linked to CRC progression and survival [40]. Here we extend these findings by showing that rosiglitazone-activated PPARg upregulates KLF4, B3GNT6, and MUC2, accompanied by enhanced mucin O-glycosylation and epithelial barrier integrity. Thus, beyond its known role in proliferation control, PPARg activation may suppress tumors by preserving mucosal barrier homeostasis via the KLF4-B3GNT6/MUC2 axis- a new dimension of PPARg’s tumor-suppressive function. Nevertheless, the direct causal relationship between PPARg activation and downstream glycosylation regulation remains to be firmly established, as our in vivo data only show association. This axis nonetheless represents a potential therapeutic target for CRC prevention and early intervention.

This study has several limitations. First, all in vivo experiments were conducted in wild-type mice using the AOM/DSS model, which does not allow direct assessment of the specific contribution of the PPARg-KLF4-B3GNT6-MUC2 axis. Intestinal epithelial cell-specific conditional knockout of Pparg, Klf4, or B3gnt6 will be required to establish definitive lineage-specific causality. Second, Core 3 O-glycosylation was assessed using BSL-II lectin staining, an indirect method with limited glycan resolution. Future application of mass spectrometry-based O-glycomics will enable precise mapping of MUC2 O-glycomics and quantification of Core 3 abundance. Third, although Core 3 glycans represent a minor fraction compared to Core 1-dominant MUC2 glycosylation, their functional impact on mucin stability and host-microbe interactions may be disproportionately significant. Consequently, more physiologically relevant models-such as gnotobiotic mice colonized with defined microbial consortia- are warranted to dissect the bidirectional crosstalk between B3GNT6-mediated glycosylation and gut microbiota. Finally, expanding clinical cohorts to integrate barrier function assays, glycomic profiles, and patient outcomes will facilitate translation of this axis into clinically actionable biomarkers or therapeutic strategies for CRC prevention.

In summary, these findings reveal that rosiglitazone activates the PPARg-KLF4-B3GNT6/MUC2 axis to restore MUC2 expression and glycosylation, preserve mucosal barrier function, and inhibit CRC progression. More broadly, this study highlights epithelial glycosylation and barrier maintenance as an underappreciated but potentially targetable dimension of tumor biology, offering new perspectives for CRC prevention and therapy.

## Materials and methods

A detailed description of all experimental procedures is provided in the Supplementary Methods section. Information on reagents, equipment, and software used in this study is listed in Suppl. Table 5.

### External data set analysis

Transcriptomic and clinical data from TCGA-COAD and GTEx were analyzed to assess gene expression patterns, survival outcomes, and pathway enrichment using publicly available platforms including LinkedOmics, DAVID, and GeneMANIA.

### Patient samples and ethical statement

Colorectal tumor and adjacent tissues (≥ 5 cm from tumor margin) were collected from 31 patients at the Third Xiangya Hospital of Central South University with informed consent and ethical approval (IRBS2023049).

### Immunohistochemistry (IHC) assay

Paraffin-embedded sections of colorectal tissues were stained with antibodies against target proteins, and DAB development was used for visualization. Quantitative image analysis was performed using ImageJ.

### Alcian blue staining

Mucin production in colon tissues was assessed by Alcian blue staining to visualize acidic polysaccharides in goblet cells.

### Lectin staining of tissue sections

BSL-II lectin histochemistry was used to detect glycosylation patterns in paraffin-embedded colon sections.

### Western blotting

Total protein was extracted from cultured cells or tissues using RIPA buffer, quantified by BCA assay, and resolved by SDS-PAGE. Proteins were transferred to PVDF membranes, incubated with specific primary and HRP-conjugated secondary antibodies, and detected using chemiluminescence. Full and uncropped western blot images are provided in the “Original Data” section.

### Lectin blotting

To assess glycosylation, proteins were transferred to PVDF membranes and probed with biotinylated Bandeiraea Simplicifolia Lectin II (BSL-II). Signal development was performed using DAB substrate for lectin-specific visualization.

### Cell culture and drug treatment

A panel of human colorectal cancer cell lines and normal epithelial cell lines was cultured under standard conditions and treated with small molecule compounds including rosiglitazone, GW9662, and kenpaullone.

### siRNA and plasmid transfection

LOVO cells were transfected with siRNAs or plasmids targeting MUC2, PPARg, KLF4 [41], and B3GNT6 to modulate gene expression.

### RT-qPCR

Total RNA was extracted using Trizol and reverse-transcribed. Gene expression was quantified using SYBR-based qPCR with gene-specific primers.

### Cell viability and clonogenic assay

Cell proliferation was measured using the Cell Counting Kit-8 (CCK-8) assay. LOVO cells were seeded in 96-well plates. For clonogenic assays, 3000 cells were seeded in 6-well plates and cultured for 14 days. Colonies were fixed with 4% paraformaldehyde and stained with crystal violet to evaluate long-term survival and proliferative capacity.

### EdU incorporation assay and quantification

Cell proliferation was assessed using the Click-iT™ EdU Imaging Kit (Thermo Fisher Scientific). EdU incorporation was quantified using ImageJ, and EdU-positive cells were defined by a bimodal Gaussian threshold derived from the control group.

### ELISA

Secreted MUC2 levels in the culture supernatant and mouse intestinal mucus were quantified using a commercially available ELISA kit. Supernatants were collected, centrifuged at 1000 g for 20 min at 2–8 °C, and processed according to the kit protocol. A standard curve was generated to calculate absolute MUC2 concentrations using Curve Expert software.

### StcE digestion assay

To evaluate mucin cleavage, cells were incubated with 5 μg/mL of the glycoprotease StcE for 2 h at 37 °C. Cell lysates were then prepared in non-denaturing lysis buffer containing Tris-Cl, NP-40, Triton X-100, glycerol, and NaCl [42]. After centrifugation, supernatants containing cleaved glycoproteins were analyzed by Western blot to assess changes in MUC2 banding patterns and molecular weight.

### Chromatin Immunoprecipitation (ChIP) assay

ChIP-qPCR was conducted to determine transcription factor occupancy at predicted regulatory regions in LOVO cells. Cells were crosslinked with 1% formaldehyde to stabilize DNA-protein interactions and quenched with glycine. After cell lysis, chromatin was sonicated into 200–500 bp fragments and incubated overnight with antibodies against target transcription factors. Immune complexes were pulled down using Protein A/G magnetic beads, and crosslinks were reversed. DNA was purified and used as template for qPCR amplification of specific promoter regions using gene-specific primers. Fold enrichment was calculated relative to input controls.

### Luciferase reporter assay

Whole cell genomic DNA was extracted from LOVO cells using a genomic DNA kit (Tiangen, DP304). Sequences spanning 2 kb upstream to 100 bp downstream of the translation start site, were amplified using a DNA polymerase kit. Amplified fragments were cloned into pGL3-Basic plasmid. The transcription factor binding site was mutated using a mutagenesis kit (Vazyme, C215-01). The pGL3-Basic plasmid or mutant plasmid containing the gene promoter sequence was co-transfected into LOVO cells with the pRL-TK plasmid, and the cells were incubated for 2 days and then assayed for luciferase activity using a dual-luciferase reporter assay system (UEBlandy, UE-F6075). The firefly luciferase activity was normalized using Renilla luciferase activity.

### Co-IP and lectin pulldown

Co-immunoprecipitation (Co-IP) was performed to evaluate protein-protein interactions in LOVO cells transfected with Flag-tagged constructs. Cell lysates were incubated with anti-Flag magnetic beads or specific antibodies (e.g., anti-MUC2, anti-B3GNT6) coupled to Protein A/G magnetic beads. After washing and elution, bound proteins were analyzed by Western blot. For lectin pulldown, membranes were probed with BSL-II lectin following immunoprecipitation to assess glycosylation-dependent binding patterns.

### Mouse models

All animal experiments were conducted in accordance with the Regulations for the Administration of Affairs Concerning Experimental Animals of China. The study protocol was approved by the Institutional Animal Care and Use Committee of Central South University (Approval No. CSU-2022-0684). Female C57BL/6 J mice were orally administered Rosiglitazone or GW9662. Colonic tissues were collected for histological and biochemical analysis.

### Xenograft tumor model

LOVO cells stably overexpressing B3GNT6 were generated using a lentiviral system, with empty vector-transduced cells as controls. Cells (5 × 10^6^) were subcutaneously injected into the flanks of female BALB/c nude mice. At the endpoint, tumors were excised, weighed, and processed for histological analysis. B3GNT6 and MUC2 expression were assessed by immunohistochemistry, and mucin-associated glycosylation was evaluated using BSL-II lectin staining.

### AOM/DSS model

A colitis-associated cancer model was established using azoxymethane (AOM) injection and repeated cycles of dextran sulfate sodium (DSS) in drinking water, followed by tumor evaluation and DAI scoring.

### Histological scoring

Colonic inflammation and tissue architecture were assessed on HE-stained sections using scoring criteria from Horino et al. [43] (see Suppl. Table S1).

### 16S rRNA sequencing

Microbial DNA from cecal contents was sequenced at the V3-V4 region to evaluate gut microbiota composition and diversity using QIIME2 and R.

### Statistical analysis

Sample sizes were determined based on previous studies in the field and our pilot experiments; no formal statistical methods were used to predetermine sample size. No samples or animals were excluded from the analysis. These criteria were defined prior to data analysis.

For cell-based experiments, samples were not randomized due to the nature of the in vitro experimental design. For animal studies, animals were randomly assigned to experimental groups where applicable.

Blinding was not applied during in vitro experiments due to the nature of the experimental design. For in vivo experiments and outcome assessment, investigators were not blinded to group allocation unless otherwise specified. Quantitative image analyses were performed using standardized and automated pipelines to minimize bias.

Two-group comparisons were performed using unpaired two-tailed Student’s t-tests, and multiple group comparisons were conducted using one-way ANOVA followed by appropriate post hoc tests. These tests were selected based on standard practices for normally distributed data. Data are presented as mean ± SD to indicate variability within each group. Normality and homogeneity of variance were assumed for parametric testing, and variance was considered similar between groups. A *p*-value < 0.05 was considered statistically significant.

## Supplementary information


Supplementary materials
Original Western Blot Data


## Data Availability

Data is available upon reasonable request. All data are available from the corresponding author upon request.
