# Supplementary material for: Restoring O-glycosylation and expression of MUC2 limits progression of colorectal cancer
Source: Cell Death Dis. 2026 Jun 21;17(1):768. doi: 10.1038/s41419-026-08981-x (PMC13530250; doi:10.1038/s41419-026-08981-x)
Supplement: Supplementary file 1 [file 41419_2026_8981_MOESM1_ESM.pdf]

Restoring O-glycosylation and expression of MUC2 limits progression of colorectal cancer

Supplementary Materials

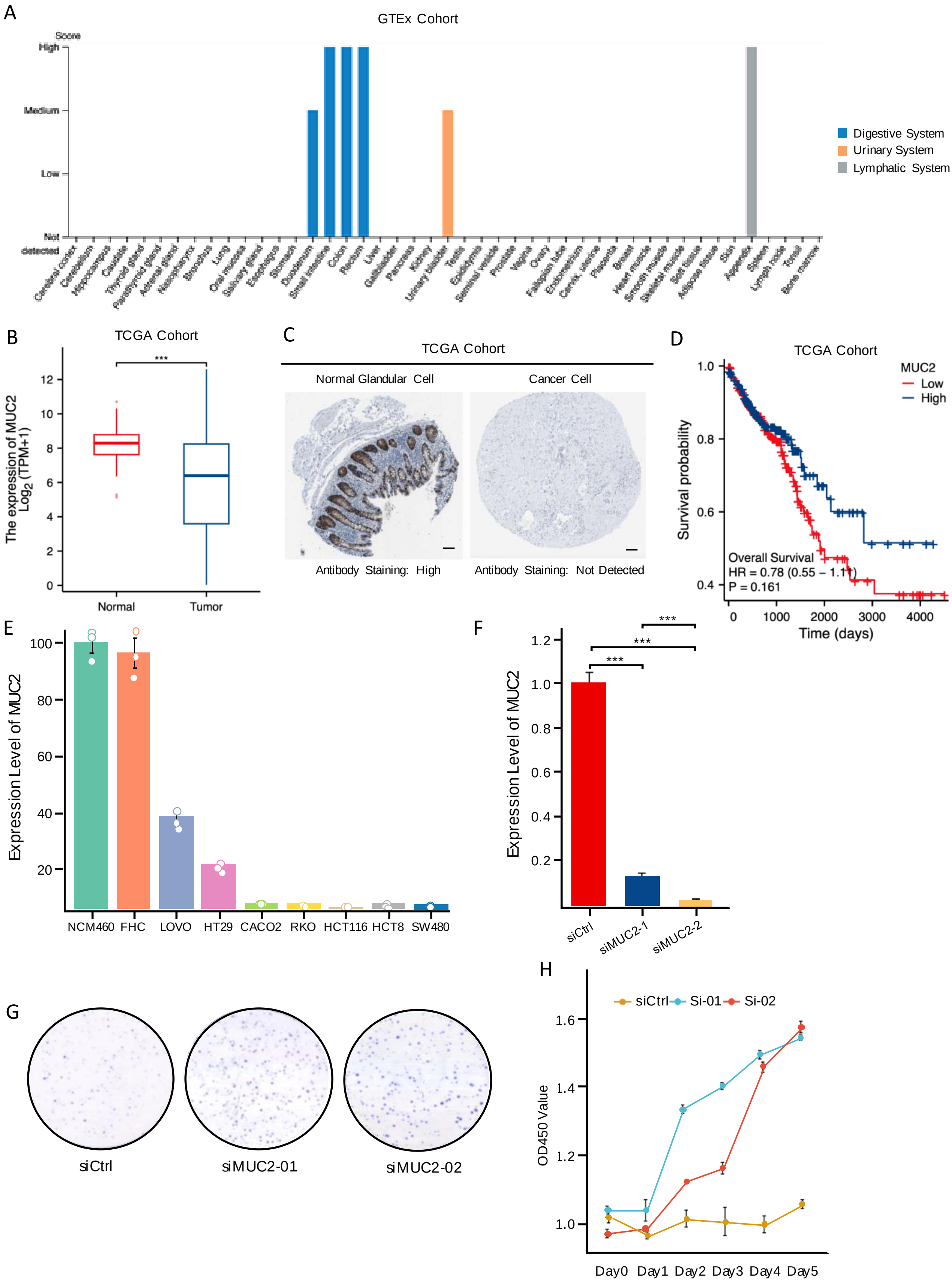

Supplementary Figure 1. MUC2 knockdown promotes proliferation of colorectal cancer cells. (A) MUC2 expression and tissue distribution in human organs from the GTEx database. (B) Boxplot of MUC2 expression in tumor versus normal tissues in TCGA-CRC cohort. (C) IHC staining of MUC2 in CRC patients from the Human Protein Atlas. Scale Bars, 200uM (D) Kaplan–Meier survival curves comparing MUC2-High and MUC2-Low CRC patients in TCGA. (E) qPCR showing relative MUC2 mRNA levels in different CRC cell lines. (F) qPCR of MUC2 expression in LOVO cells transfected with siRNAs (n=3, Ctrl; n=3, siMUC2-1; n=3, siMUC2-2). (G) Colony formation assay of LOVO cells after MUC2 knockdown (n=3 per group). (H) Cell proliferation assay (CCK-8) in LOVO cells after siRNA transfection (n=5 per group). Data were analyzed by one-way ANOVA with Tukey's post-hoc test. Error bars represent mean ± SEM. *$p<0.05$, **$p<0.01$, ***$p<0.001$. qPCR, quantitative polymerase chain reaction; IHC, immunohistochemistry; CCK-8, Cell Counting Kit-8; CRC, colorectal cancer. cell counting kit-8.

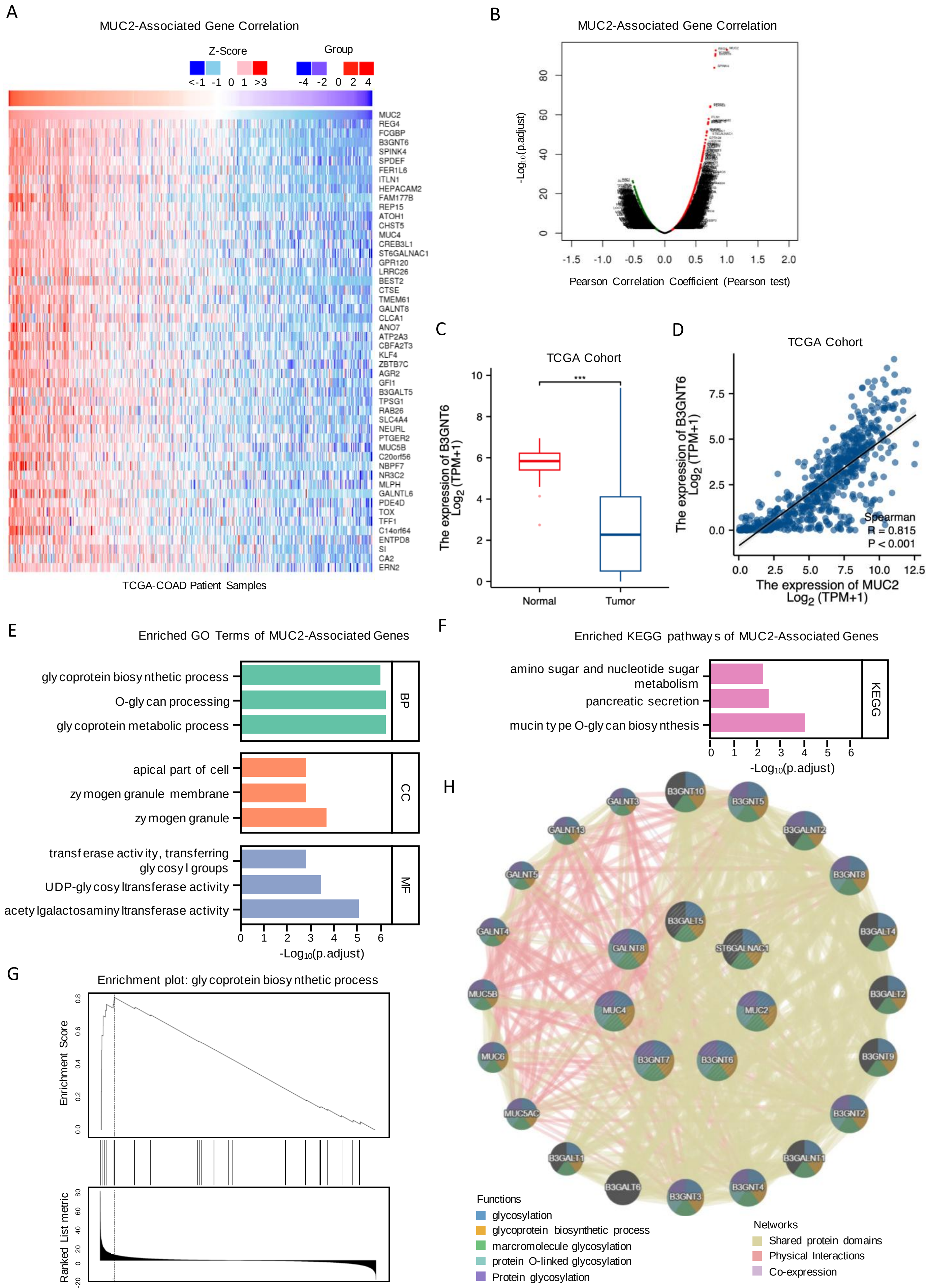

Supplementary Figure 2. B3GNT6 is a key molecule in the MUC2 O-glycosylation pathway. (A–B) Correlation between MUC2 and other genes in TCGA dataset visualized by heatmap and volcano plot. Red/blue dots indicate significantly positive/negative correlations, respectively. (C) Boxplot comparing B3GNT6 expression levels in tumor and normal tissues in TCGA-CRC cohort. (D) Scatter plot showing a positive Pearson correlation between MUC2 and B3GNT6 expression in TCGA. CRC, colorectal cancer. (E) GO analysis showing biological processes enriched among genes associated with MUC2. (F) KEGG pathway analysis of genes correlated with MUC2. (G) GSEA showing enrichment of the Mucin-type O-glycan biosynthesis pathway among MUC2-associated genes. (H) PPI network constructed using GeneMANIA based on MUC2-related genes. GO, Gene Ontology; KEGG, Kyoto Encyclopedia of Genes and Genomes; GSEA, Gene Set Enrichment Analysis; PPI, protein–protein interaction; CRC, colorectal cancer.

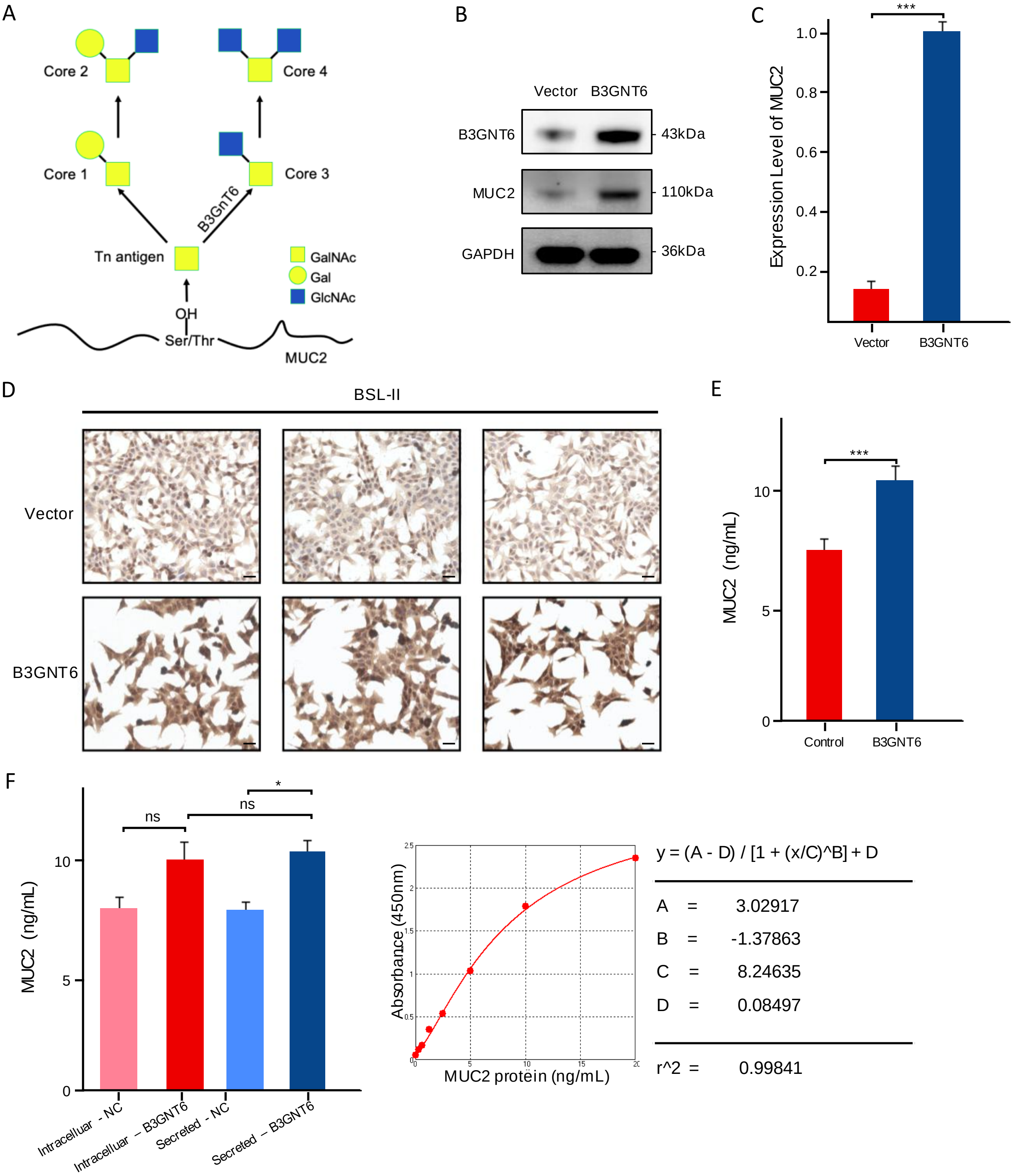

Supplementary Figure 3. B3GNT6 enhances MUC2 secretion and Core3 glycosylation. (A) Schematic of O-glycosylation biosynthesis pathway. (B-C) qPCR and Western blot showing MUC2 upregulation in LOVO cells after B3GNT6 overexpression (n=3, Ctrl; n=3, B3GNT6-OE). (D) BSL-II lectin staining of Core3 in LOVO cells after B3GNT6 (n=3, Ctrl; n=3, B3GNT6-OE). Scale bars, 50 μm. (E) ELISA measuring MUC2 secretion into the supernatant (n=3, Ctrl; n=3, B3GNT6-OE). (F) ELISA detecting intracellular and secreted MUC2 levels in LOVO cells (n=5, Intracellular-Ctrl; n=5, Intracellular-B3GNT6; n=5, Secreted-Ctrl; n=5, Secreted-B3GNT6). Data were analyzed by one-way ANOVA with Tukey's post-hoc test. Error bars represent mean ± SEM. *p<0.05, **p<0.01, ***p<0.001. ELISA, enzyme-linked immunosorbent assay; OE, overexpression; CRC, colorectal cancer.

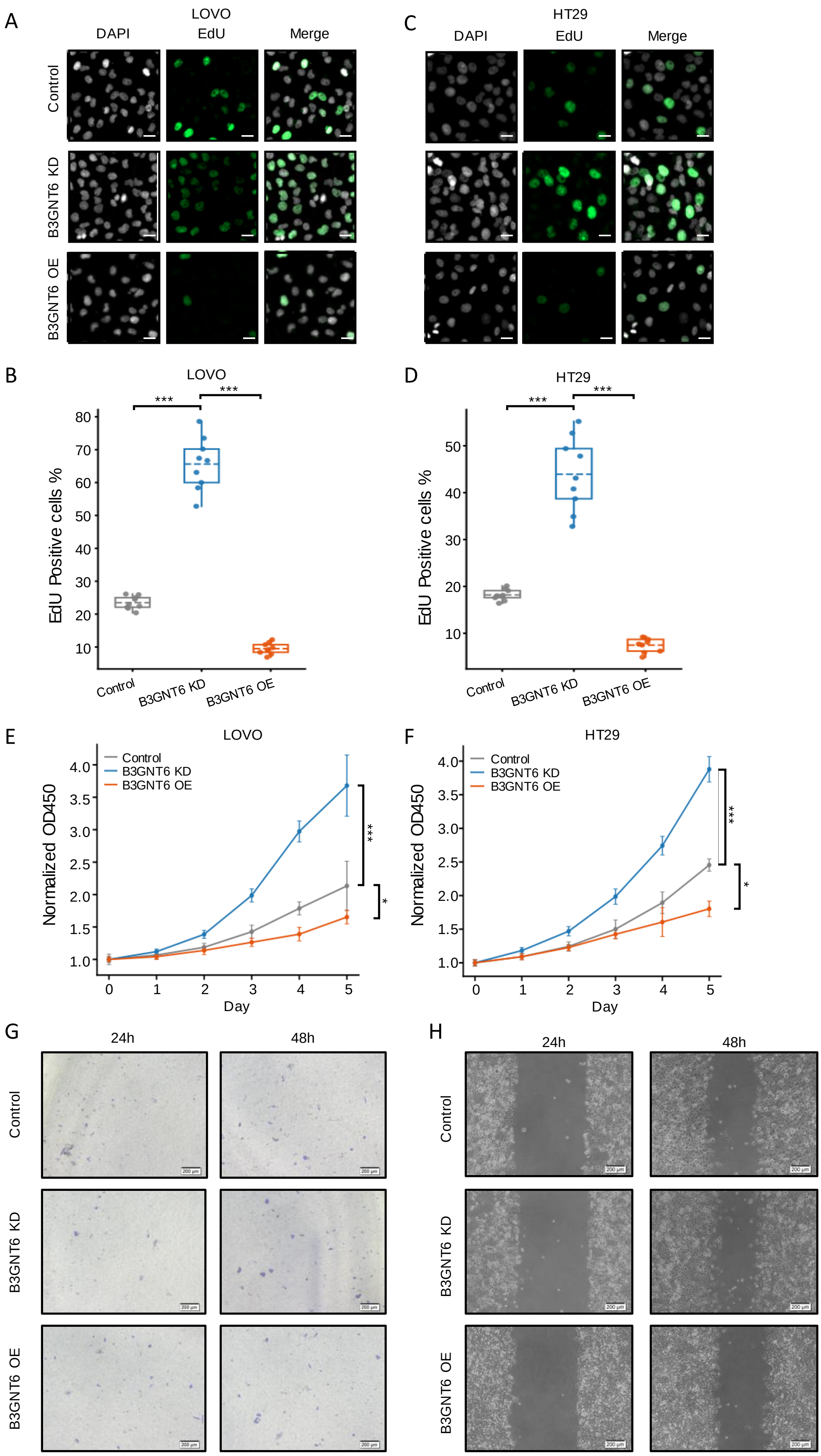

Supplementary Figure 4. B3GNT6 suppresses CRC cell proliferation in vitro. (A) Representative fluorescence images of EdU incorporation in LOVO cells with B3GNT6 knockdown or overexpression. Scale bars, 20 μm. (B) Quantification of EdU-positive cells in LOVO cells. (C) Representative fluorescence images of EdU incorporation in HT29 cells with B3GNT6 knockdown or overexpression. Scale bars, 20 μm. (D) Quantification of EdU-positive cells in HT29 cells. (E) Cell proliferation curves of LOVO cells with B3GNT6 knockdown or overexpression measured by CCK-8 assay. (F) Cell proliferation curves of HT29 cells with B3GNT6 knockdown or overexpression measured by CCK-8 assay. (G) Wound healing assay showing cell migration ability. (H) Transwell assay evaluating cell invasion. Scale bars, 200 μm. Data are presented as mean ± SEM. Statistical significance was determined using unpaired two-tailed Student's t-test. $^*P < 0.05$, $^{**}P < 0.01$, $^{***}P < 0.001$.

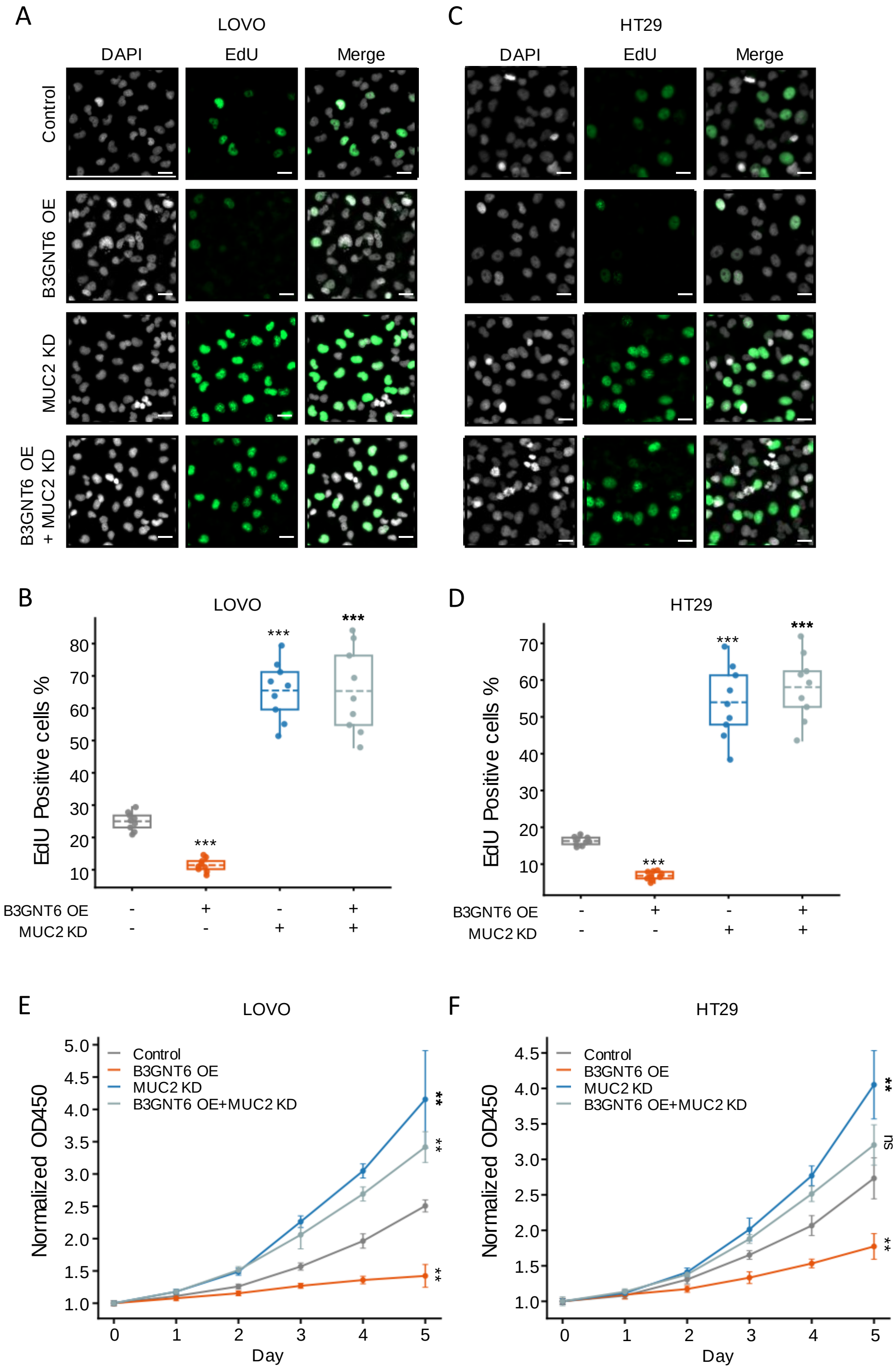

Supplementary Figure 5. The anti-proliferative effect of B3GNT6 depends on MUC2 in CRC cells. (A) Representative fluorescence images of EdU incorporation in LOVO cells with B3GNT6 overexpression alone or combined with MUC2 knockdown. (B) Quantification of EdU-positive cells in LOVO cells. (C) Representative fluorescence images of EdU incorporation in HT29 cells under the same conditions. (D) Quantification of EdU-positive cells in HT29 cells. (E) Cell proliferation curves of LOVO cells with B3GNT6 overexpression alone or combined with MUC2 knockdown measured by CCK-8 assay. (F) Cell proliferation curves of HT29 cells under the same conditions measured by CCK-8 assay. Scale bars, 20 μm. Data are presented as mean ± SEM. Statistical significance was determined using unpaired two-tailed Student's t-test, with comparisons made against the Control group. *P < 0.05, **P < 0.01, ***P < 0.001.

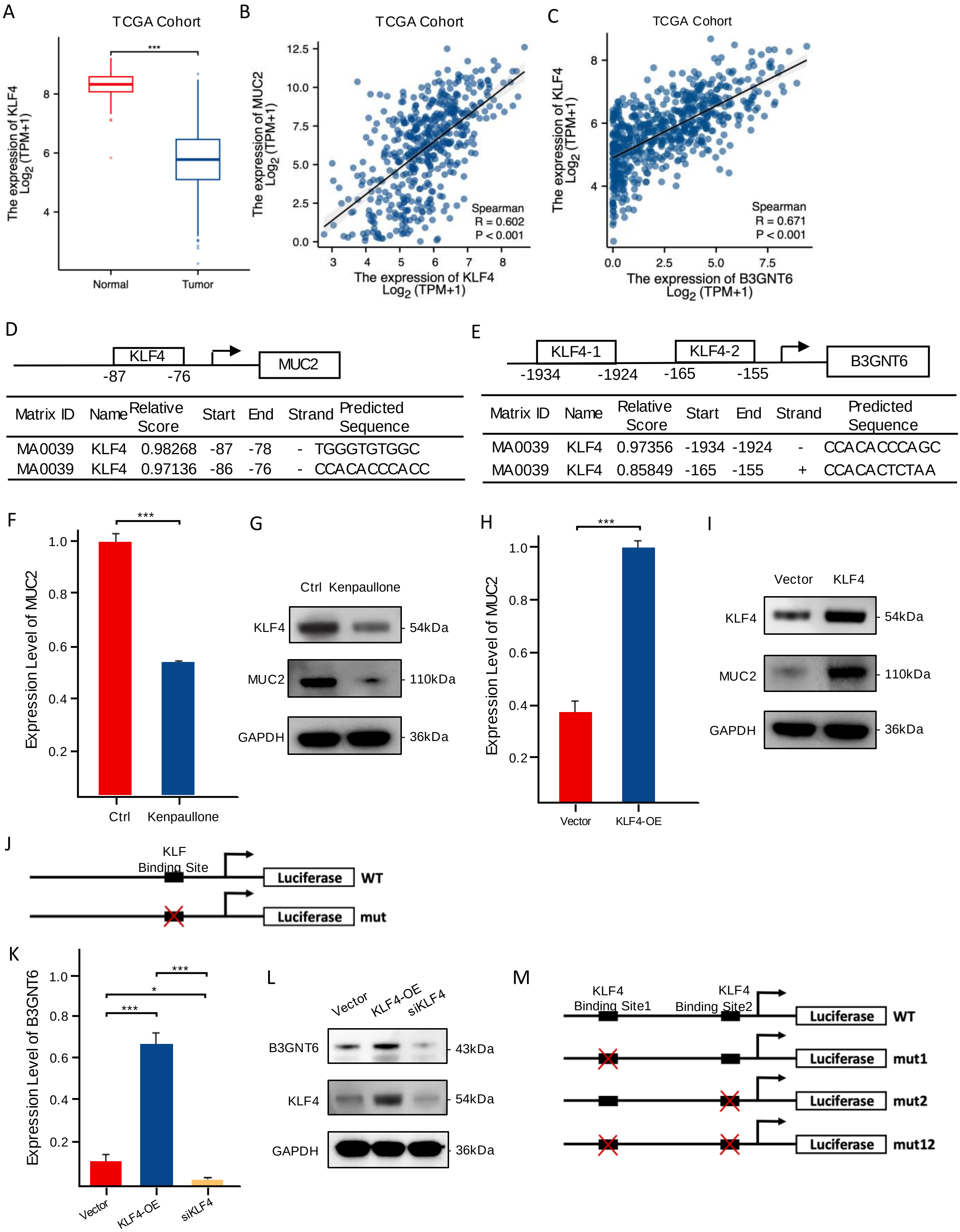

Matrix ID	Name	Relative Score	Start	End	Strand	Predicted Sequence
MA0039	KLF4	0.98268	-87	-78	-	TGGGTGTGGC
MA0039	KLF4	0.97136	-86	-76	-	CCACACCCACC

Matrix ID	Name	Relative Score	Start	End	Strand	Predicted Sequence
MA0039	KLF4	0.97356	-1934	-1924	-	CCACACCCAGC
MA0039	KLF4	0.85849	-165	-155	+	CCACACTCTAA

Supplementary Figure 6. KLF4 regulates MUC2 and B3GNT6 transcription. (A) Boxplot of KLF4 expression in TCGA-CRC cohort (tumor vs. normal). (B) Correlation plot showing positive association between MUC2 and KLF4 expression. (C) Correlation between B3GNT6 and KLF4 in TCGA-CRC dataset. (D-E) Schematic diagrams of KLF4 binding site in MUC2 and B3GNT6 promoters. (F-G) qPCR and Western blot of MUC2 after KLF4 inhibitor Kenpaullone (2.5 μM) treatment (n=3, Ctrl; n=3, Kenpaullone). (H-I) qPCR and Western blot of MUC2 in LOVO cells after KLF4 overexpression cells (n=3, Ctrl; n=3, KLF4-OE). (J) Schematic diagrams of KLF4 binding site in MUC2 WT/mutant luciferase constructs. (K-L) qPCR and Western blot of B3GNT6 after KLF4 overexpression or knockdown (n=3, Ctrl; n=3, KLF4-OE; n=3, siRNA-KLF4). (M) Diagram of WT and mutant B3GNT6 promoter luciferase constructs (mut1: 1924–1934 bp; mut2: 155–165 bp). Data were analyzed by one-way ANOVA with Tukey's post-hoc test. Error bars represent mean ± SEM. *p<0.05, **p<0.01, ***p<0.001. qPCR, quantitative polymerase chain reaction; CRC, colorectal cancer.

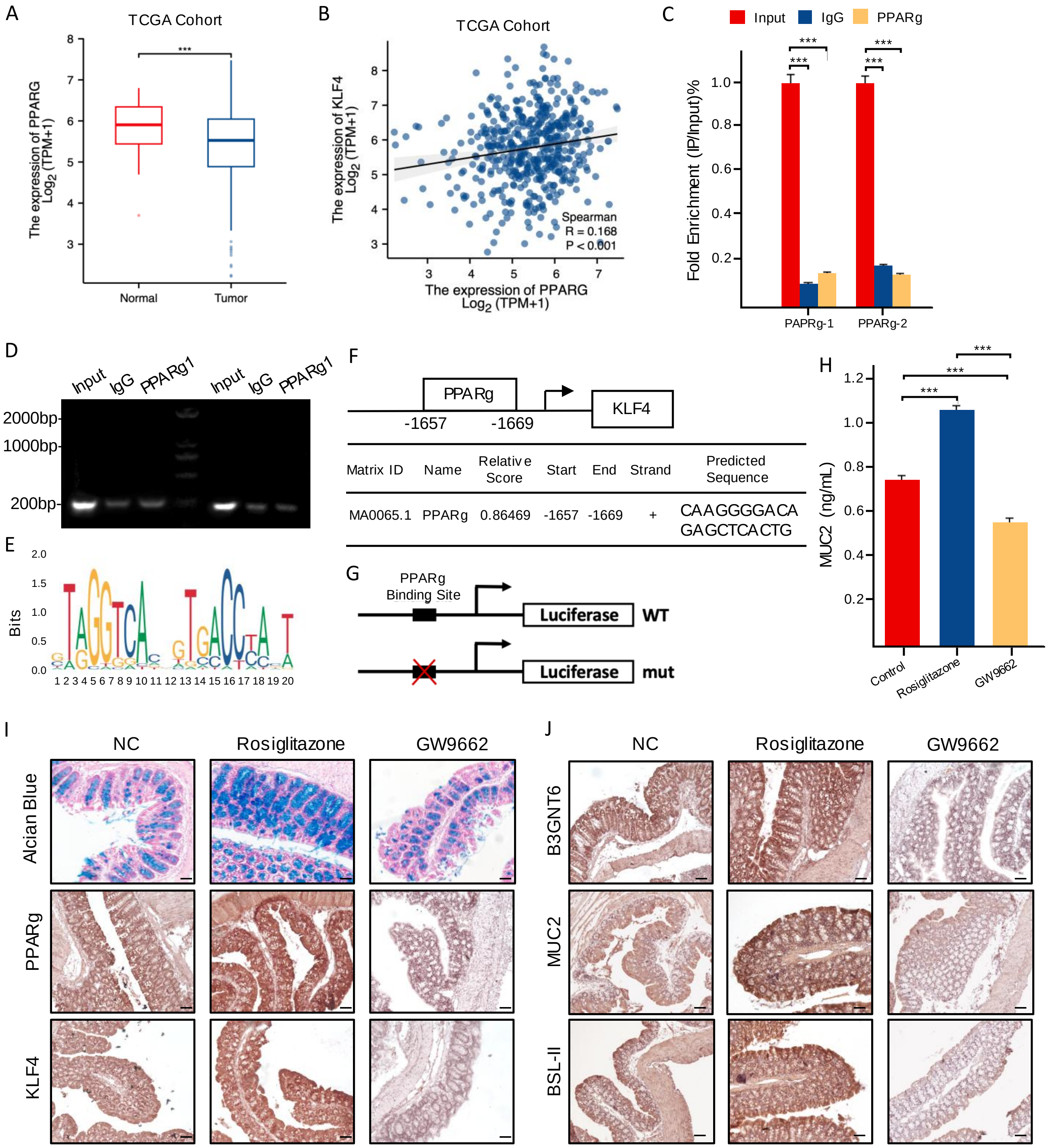

Supplementary Figure 7. Prediction of PPARg binding sites in MUC2 and KLF4 promoter regions. (A) Boxplot of PPARg expression in TCGA-CRC cohort. (B) Positive correlation between PPARg and KLF4 in TCGA. (C-D) ChIP-qPCR identifying PPARg binding at site 1 (–1959 to –1969 bp) and site 2 (–517 to –535 bp) relative to the ATG start codon (n=3, Input; n=3, IgG; n=3, CHIP-PPARg). (E) Sequence logo of PPARg binding motifs from the JASPAR database. (F) Diagram of PPARg binding regions in the MUC2 promoter. (G) Schematic of KLF4 promoter constructs used in luciferase assay (WT and mutant). (H) ELISA measuring MUC2 levels in supernatant after treatment with Rosiglitazone (40 µM) or GW9662 (20 µM) (n=5, Ctrl; n-5, Rosiglitazone; n=5, GW9662). (I-J) H&E and IHC staining of PPARg, KLF4, B3GNT6, MUC2, and BSL-II lectin staining of Core3 in C57BL/6 mice treated with Rosiglitazone or GW9662 (10 mg/kg) (n=3, Ctrl; n=3, Rosiglitazone; n=3, GW9662). Scale bars, 50 µm. Data were analyzed by one-way ANOVA with Tukey's post-hoc test. Error bars represent mean ± SEM. *p<0.05, **p<0.01, ***p<0.001. ANOVA, analysis of variance; H&E, hematoxylin-eosin; IHC, immunohistochemistry; ELISA, enzyme-linked immunosorbent assay; CRC, colorectal cancer.

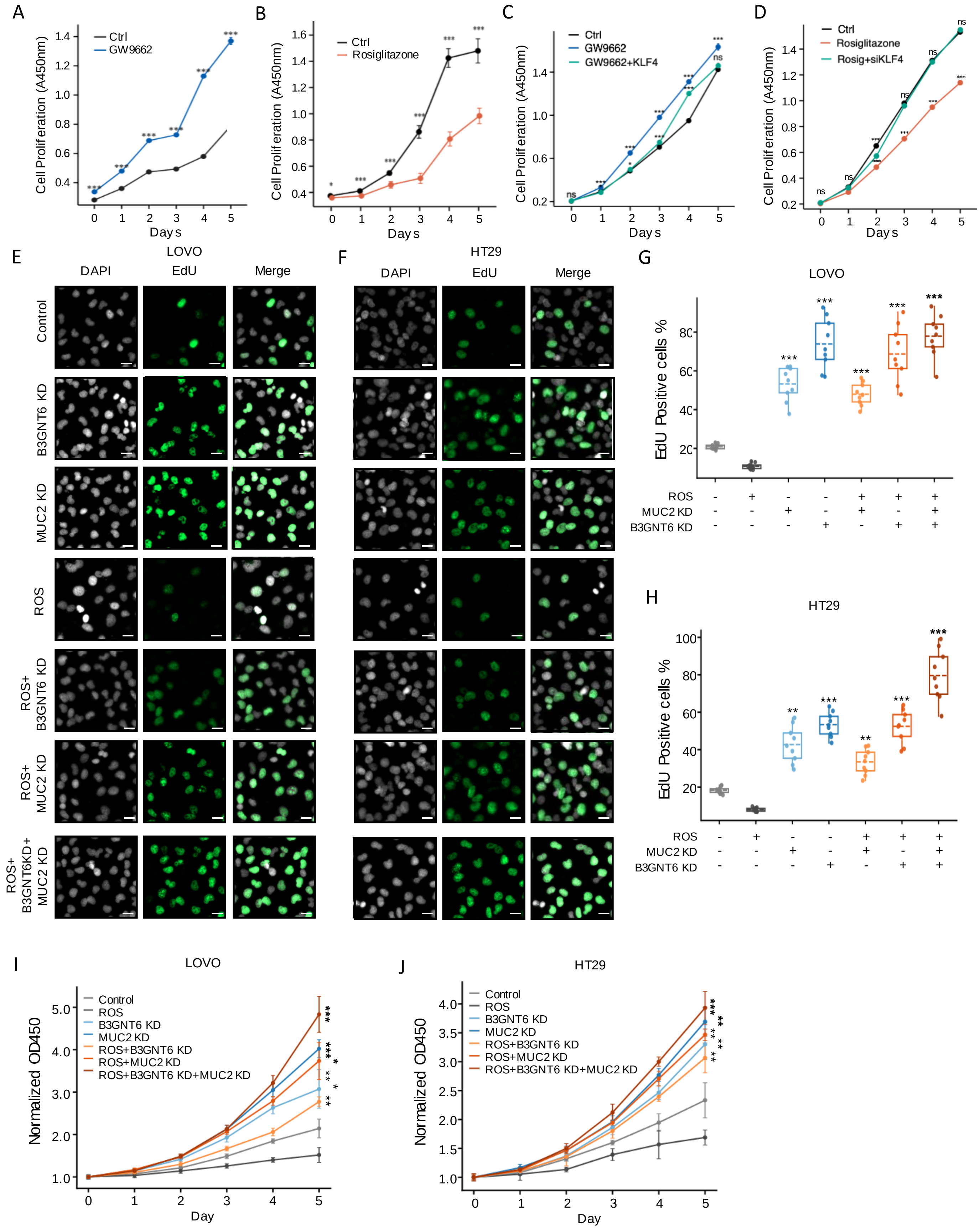

Supplementary Figure 8. The anti-proliferative effect of rosiglitazone depends on the B3GNT6–MUC2 axis in CRC cells. (A–B) CCK-8 assay measuring cell proliferation in LOVO cells treated with Rosiglitazone (40 μM) or GW9662 (20 μM). (C–D) CCK-8 assay after KLF4 overexpression + GW9662 or KLF4 knockdown + Rosiglitazone treatment. (E-F) Representative fluorescence images of EdU incorporation in LOVO and HT29 cells treated with rosiglitazone (ROS) alone, ROS combined with B3GNT6 knockdown, or ROS combined with both B3GNT6 and MUC2 knockdown. (G-H) Quantification of EdU-positive cells in LOVO and HT29 cells under the indicated conditions. (I) Cell proliferation curves of LOVO cells under the indicated treatments measured by CCK-8 assay. (J) Cell proliferation curves of HT29 cells under the indicated treatments measured by CCK-8 assay. Scale bars, 20 μm. Data are presented as mean ± SEM. Statistical significance was determined using unpaired two-tailed Student's t-test, with comparisons made against the rosiglitazone treated group (ROS). $*P < 0.05$, $**P < 0.01$, $***P < 0.001$.

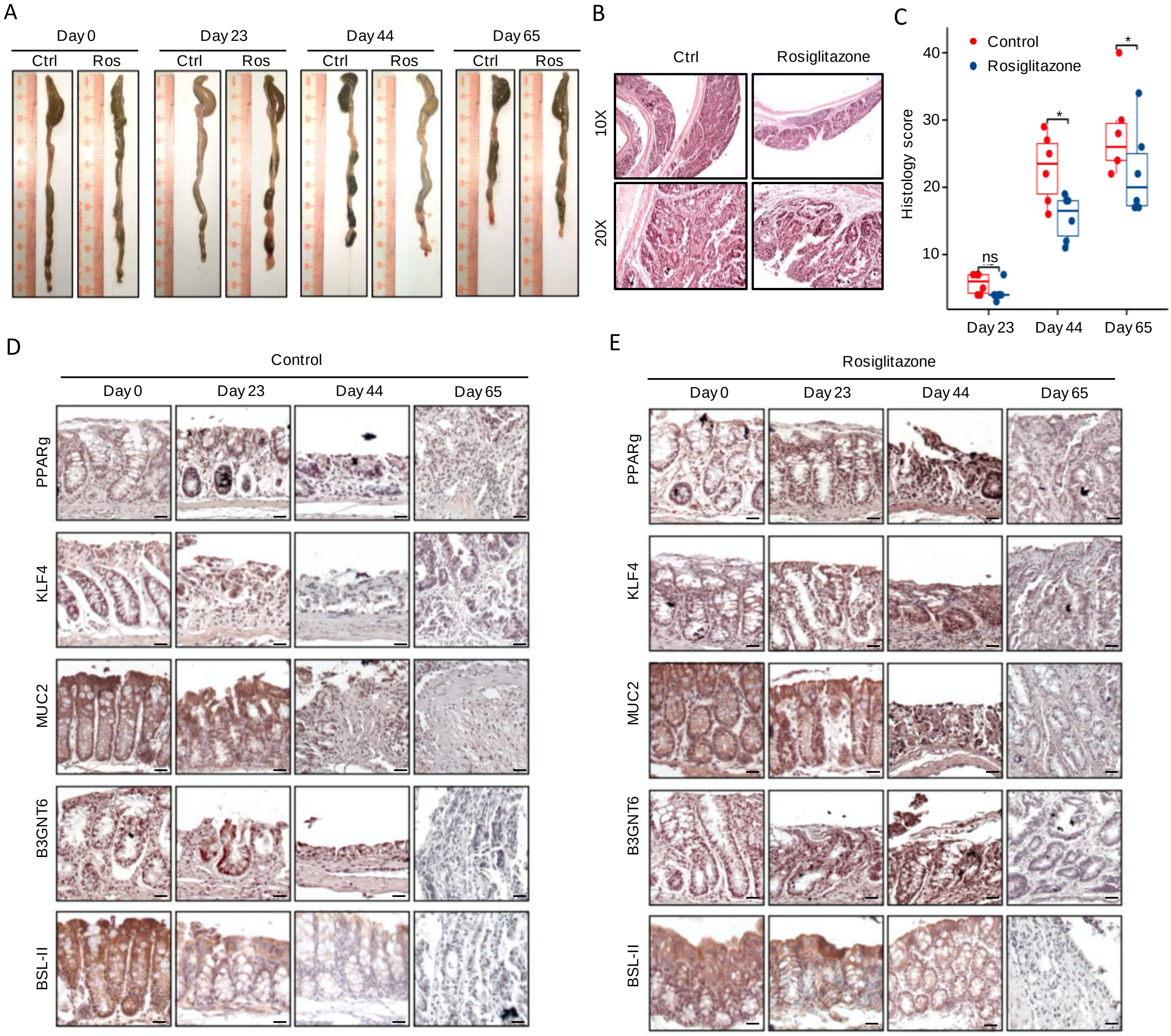

Supplementary Figure 9. Rosiglitazone inhibits proliferation of colorectal cancer cells. (A) Representative images of colons at different stages of AOM/DSS treatment. (B-C) H&E staining and histology of colon tissues. Scale bars, 200 μm. (D-E) IHC staining for PPARg, KLF4, MUC2, B3GNT6 and BSL-II of intestinal mucus in mouse colonic tissues at four stages (Day 0, Day 23, Day 44, Day 65 of DSS cycle. (n=6, AOM/DSS-Control; n=6, AOM/DSS-Rosiglitazone). Scale bars, 50 μm. Data were analyzed by one-way ANOVA with Tukey's post-hoc test. Error bars represent mean ± SEM. *p<0.05, **p<0.01, ***p<0.001. CCK-8, Cell Counting Kit-8; IHC, immunohistochemistry; CRC, colorectal cancer.

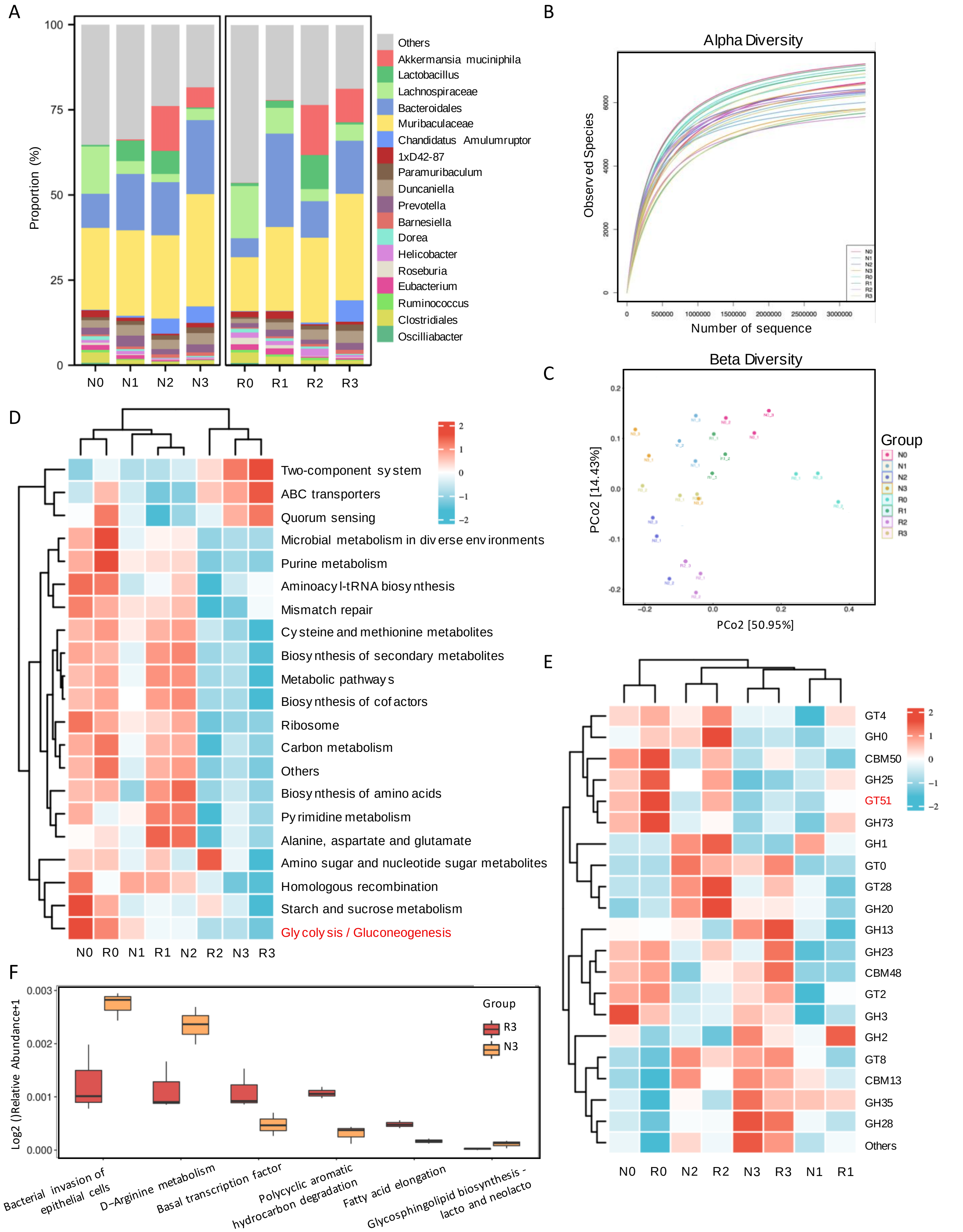

Supplementary Figure 10. Rosiglitazone enhances glycosylation-related microbial functions and suppresses bacterial invasion in AOM/DSS-induced colorectal cancer model. (A) Stacked bar chart showing changes in microbial composition during CRC progression in mice treated with or without Rosiglitazone (N: AOM/DSS-Control group; R: AOM/DSS-Rosiglitazone group). (B) Rarefaction curves reflecting alpha diversity of gut microbiota. (C) Principal Coordinates Analysis (PCoA) illustrating beta diversity shifts. (D) Heatmap showing expression levels of functional protein sequences from gut microbiota, annotated via Unigene database using DIAMOND software against the KEGG reference. (E) Heatmap showing carbohydrate-active enzyme gene annotation based on the CAZy database, indicating enhanced glycosylation capacity in the Rosiglitazone-treated group. (F) KEGG pathway enrichment analysis of microbial genes in tumor-bearing mice. Statistical tests were performed within the corresponding microbial analysis pipelines. KEGG, Kyoto Encyclopedia of Genes and Genomes; CAZy, Carbohydrate-Active enZYmes; PCoA, Principal Coordinates Analysis.

Table S1. Histology Score

Feature scored	Score	Description
Inflammation severity	0	None
	1	Mild
	2	Moderate
	3	Severe
Inflammation extent	0	None
	1	Mucosa
	2	Mucosa and sub-mucosa
	3	Transmural
Crypt damage	0	None
	1	Basal 1/3 damaged
	2	Basal 2/3 damaged
	3	Crypts lost; surface epithelium present
	4	Crypts and surface epithelium lost
Percent involvement(%)	0	0
	1	1-25
	2	26-50
	3	51-75
	4	76-100

Table S2. Antibodies

Antibodies	Source	Identifier	WB	IHC
MUC2 Rabbit pAb	Abclonal	A14659	1:1000	1:200
KLF4 Polyclonal antibody	Proteitech	11880-1-AP	1:1000	1:300
PPAR Gamma Polyclonal antibody	Proteitech	16643-1-AP	1:5000	1:300
Rabbit anti-Homo sapiens (Human) B3GNT6 Polyclonal antibody	Cusabio	CSB-PA744392LA01HU	1:3000	1:300
Rabbit anti-Homo sapiens (Human) KI67 Polyclonal antibody	Cusabio	CSB-PA11617A0Rb	——	1:300
GAPDH Monoclonal antibody	Proteitech	60004-1-Ig	1:10000	——

Table S3. Primers

Genes	NCBI Reference Sequence	Sequence-F(5'-3')	Sequence-R (3'-5')
MUC2	NM_002457.5	GACGGCGATGTCTACCGATT	GGTCAGCAGCCTCTCACATT
KLF4	NM_001314052.2	GTCCCGGGGATTTGTAGCTC	TGTAGTGCTTTCTGGCTGGG
PPARg	NM_001330615.4	GCCTTAACCTCTGCTGGTGA	GGAGATGCAGGCTCCACTTT
B3GNT6	NM_138706.5	ATCCAGGACTTCCTGCGGTA	CGGGGTGCCCAATAGAAAG A
ACTB	NM_001101.5	CTCCATCCTGGCCTCGCTGT	GCTGTCACCTTCACCGTTCC

Table S4. Primers for ChIP

Genes	TF	Binding Site (upstream of ATG)	Sequence-F(5'-3')	Sequence-R (3'-5')
MUC2	KLF4	76bp-87bp	TCATCCTGAAGAAGGC TGCG	CCTGTGTCTGCTCTCT CACC
MUC2	PPARg	515bp-535bp	GCTCAGCCTGGGAAAA GGAT	GAGGACCAACCCATTC CTGG
MUC2	PPARg	1959bp-1969bp	CGTTTACCCAGGGAGC CATAA	GAGCCCCCTGCCAAA GACA
B3GNT6	KLF4	1924bp-1934bp	GTCACTGGTGGCTAAG GAAGC	CCAGCTTACCTGCAGT CTCTG
B3GNT6	KLF4	155bp-165bp	GGTGGCTAAGGAAGC CTGTA	CCAGCCTGAGACTAGC CTTT
KLF4	PPARg	1657bp-1669bp	ACATTTAAGTGTATGT GTGCTTTGA	TGGCAGTTCCTTCTAG GGTT

Table S5. Reagents, Equipment, and Software

**Reagents**		
Item	Brand	Catalog Number
Rosiglitazone	Meilunbio	MB1211
GW9662	MCE	HY-16578
Kenpaullone	Selleck	S7919
BSL-II Lectin	Vectorlabs	B-1215-2
Trizol	Beyotime	R0016
JetPRIME transfection reagent	Polyplus	101000046
Phanta Max Super-Fidelity DNA Polymerase	Vazyme	P505-d1
ChamQ Blue Universal SYBR qPCR Master Mix	Vazyme	Q312-02
HiScript IV All-in-One Ultra RT SuperMix for qPCR	Vazyme	R433-01
2 × Rapid Taq Plus Master Mix (Dye Plus)	Vazyme	P223-01
Mut Express Universal Fast Mutagenesis Kit	Vazyme	C216-01
SDS-PAGE protein electrophoresis buffer	New Cell & Molecular Biotech	WB52001
Rapid Transfer Buffer	New Cell & Molecular Biotech	WB4600
RIPA Buffer	New Cell & Molecular Biotech	WB3100
PVDF membrane	Millipore	IPVH00010
DMEM (Dulbecco's Modified Eagle Medium) cell culture media	BasalMedia	L110KJ
RPMI 1640 Cell Culture Media	BasalMedia	L220KJ
Fetal Bovine Serum (FBS)	Gibco	A5670701
QIAprep Spin Miniprep Kit	Qiagen	27104
Enhanced Chemiluminescent Kit	New Cell & Molecular Biotech	P2300
DAB Kit	ZSGB Biiotech	ZLI-9018
MUC2 ELISA Kit	Elabscience	E-EL-H0632c
Alcian Blue Stain Kit	Bioss	S0134
ChIP Assay Kit	Beyotime	P2078
**Equipment**		
Zeiss Microscope	Zeiss	Axioscope 5
LC-MS/MS	Thermo Scientific	LTQ Orbitrap Velos Pro ETD
Gel Imaging System	Bio-Rad	ChemiDoc XRS+
Thermal Cycler	Bio-Rad	T100
Real-Time PCR	Bio-Rad	CFX96 Touch
Electrophoresis System	Bio-Rad	Mini-PROTEAN®
Blotting System	Bio-Rad	Mini Trans-Blot® Cell
**Software**		
GraphPad Prism	GraphPad	v7.0
ImageJ	NIH	-

Materials and methods

External Data Set Analysis

RNA-seq data from the TCGA-COAD project were downloaded in TPM format from the GDC Data Portal (https://portal. fixed in 4% paraformaldehyde for 24 hours at room temperature, followed by sequential dehydration in gradegdc.cancer.gov). Survival analysis was conducted using the Kaplan-Meier method and log-rank test.Spearman correlation between MUC2 and other genes was evaluated using LinkedOmics (http://www.linkedomics.org/).The top 100 positively or negatively correlated genes were subjected to GO and KEGG enrichment analysis using DAVID. Protein-protein interaction (PPI) networks were analyzed via GeneMANIA (http://genemania.org).

Patient Samples

Colorectal tumor and paired adjacent tissues (>=5 cm from tumor edge) were obtained from 31 colorectal cancer patients at the Third Xiangya Hospital, Central South University. All patients signed informed consent, and the study was approved by the Ethics Committee of the Third Xiangya Hospital of Central South University (ID: IRBS2023049). All patients had not received palliative surgery, chemotherapy or radiotherapy before radical surgery, and all had been confirmed by postoperative pathological examination.

Immunohistochemistry (IHC) assay

Colorectal tissue specimens were d ethanol solutions (70%, 85%, 95%, and 100%), clearing in xylene, and embedding in paraffin. Tissue blocks were sectioned at a thickness of 4 µm using a rotary microtome. Paraffin sections were deparaffinized in xylene, rehydrated through a descending ethanol series, and subjected to antigen retrieval in sodium citrate buffer (pH 6.0) using a microwave oven for 10 minutes. Endogenous peroxidase activity was quenched by incubating the slides in 3% hydrogen peroxide for 10 minutes at room temperature. After blocking in 5% BSA for 30 minutes, sections were incubated overnight at 4°C with primary antibodies diluted in blocking buffer. On the following day, slides were washed and incubated with HRP-conjugated secondary antibodies for 1 hour at room temperature. Visualization was achieved with a DAB substrate kit according to the manufacturer's protocol. Counterstaining was performed using hematoxylin. The stained slides were imaged using a Zeiss Axioscope 5 microscope, and quantitative analysis of staining intensity and area was performed using the IHC Profiler plugin in ImageJ software (NIH, Bethesda, MD, USA), assigning scores from 0 (negative) to 3+ (high positive) based on standardized thresholds.

Alcian Blue staining

To show the structure of mucus secreted by cupped cells in colonic tissue, paraffin-embedded colon sections were deparaffinized, rehydrated, and stained using an Alcian Blue staining kit according to the manufacturer's instructions (Bioss, S0134). Slides were washed, dehydrated, cleared, and mounted with neutral balsam. Images were acquired with a Zeiss Axioscope 5 microscope, and the stained area was quantified using ImageJ.

Lectin staining of Tissue sections

Lectin histochemistry was conducted to assess glycosylation patterns. After standard deparaffinization, antigen retrieval, and blocking (as described in the IHC assay), sections were incubated overnight at 4°C with Bandeiraea Simplicifolia Lectin II (BSL-II; Vectorlabs, B-1215-2) (1:300 dilution in PBS) and washed three times with PBS for 3 min each. Prepare SABC working solution according to the instructions of the SABC-HRP kit (Beyotime, P0603) and was dropped on slides and incubated for 1h at room temperature. Prepare DAB working solution according to the instructions of DAB Staining Kit (ZSGB-BIO, ZLI-9018), and incubate the slides for 10 min at room temperature. Dehydration and sealing were performed according to the experimental protocols in IHC. Imaging was performed using a microscope image system (Zeiss, Axioscope 5). The staining in the IHC images was analyzed and scored by the IHC Profiler plug-in in ImageJ software (Maryland, USA). Images were scored according to staining intensity versus staining area, High positive (3+), Positive (2+), Low Positive (1+) and Negative (0).

Western Blots (WB) analysis

Cells or tissues were lysed in RIPA buffer (Beyotime, P0013C) with protease inhibitors. Protein concentrations were quantified using a BCA Protein Assay Kit (New Cell & Molecular Biotech, WB6501). Equal amounts of protein were resolved on ExpressCast PAGE Color Gel (New Cell & Molecular Biotech, P2012) and transferred to PVDF membranes (Millipore, IPVH00010) using a LIUYI DYY-7C transfer unit at 300 mA for 2 h. Membranes were blocked with 2.5% milk in TBST, incubated with primary antibodies overnight at 4°C, and with HRP-conjugated goat anti-mouse IgG (Proteintech, PR30011) for 1 h at 37°C. Detection was performed using ECL (New Cell & Molecular Biotech, P2300). For lectin blotting, membranes were incubated with BSL-II and developed using DAB substrate. Full and uncropped western blot images are provided in the "Original Data" section.

Cell culture and Drug treatment

Human colorectal cancer cell lines (LOVO, HT29, CACO2, RKO, HCT116, HCT8, SW480) and normal colonic epithelial cell lines (NCM460 and FHC) were cultured in DMEM (Biological Industries) supplemented with 10% FBS (Gibco), 100 U/mL penicillin, and 100 µg/mL streptomycin at 37°C with 5% CO_2. Cell lines were obtained from Cancer Research Institute of Central South University. Cell line authentication was not recently performed by STR profiling. All cell lines were routinely tested and confirmed to be free of mycoplasma contamination. LOVO cells were treated with 40 µM Rosiglitazone (Meilunbio, MB1211), 20 µM GW9662 (MCE, HY-16578), or 2.5 µM Kenpaullone (Selleck, S7919) for 24 h.

Plasmids and siRNA Transfection
LOVO cells were seeded in six-well plates. When cells reached 30–50% confluence, transfection was performed using JetPRIME reagent (Polyplus) according to the manufacturer's protocol. siRNAs targeting MUC2, PPARg (RIBOBIO), and KLF4 [15] and pcDNA3.1-3xFlag-tagged plasmids for PPARg (NM_015869), KLF4 (NM_001314052), and B3GNT6 (NM_138706) were used. Cells were harvested 48 h after transfection for RNA and protein extraction.

RNA Isolation and Real-time Quantitative PCR
The cells cultured in six-well plates were first washed thoroughly with PBS. One milliliter of Trizol reagent (Beyotime, R0016) was added to each well, and the cells were lysed for 5–10 minutes at room temperature. After complete lysis, 200 µL of chloroform was added to each sample, followed by vigorous shaking and centrifugation at 4°C for 15 minutes at 12,000 × g. The aqueous upper phase was carefully transferred to a new tube, and an equal volume of isopropanol was added. The mixture was incubated at room temperature for 10 minutes and then centrifuged again at 4°C for 10 minutes at 12,000 × g to precipitate the RNA. The RNA pellet was washed twice with 75% ethanol and centrifuged at 4°C for 7 minutes at 7,500 × g. The resulting pellet was air-dried and resuspended in 30–100 µL of RNase-free water. RNA concentration and purity were assessed using a NanoDrop spectrophotometer. Reverse transcription was carried out using the HiScript II qRT SuperMix for qPCR Kit (Vazyme, R223-01) following the manufacturer's protocol. Real-time quantitative PCR was performed using the SYBR qPCR Master Mix Kit (Vazyme, Q321-02) on a StepOnePlus Real-Time PCR System (Applied Biosystems). Primer sequences used in the analysis are listed in Supplementary Table 3.

Cell viability Assay and Clonogenic Assay
LOVO cells were seeded into 96-well plates with 8000 cells per well and incubated for 12 hours. Cell Counting Kit-8 (CCK8; New Cell & Molecular Biotech, C6005) was added after 0 h, 24 h, 48 h, 72 h, 96 h, and 120 h, respectively, and the absorbance at 450nm wavelength was measured by a microplate reader after 2 h of incubation at 37°C to analyze the change in cell proliferation. For colony formation assays, 3000 cells were seeded in 6-well plates and cultured for 14 days. Colonies were fixed in 4% paraformaldehyde and stained with crystal violet (Beyotime, C0121).

EdU incorporation assay and quantification
Cell proliferation was assessed using the Click-iT EdU Imaging Kit (Thermo Fisher Scientific) following the manufacturer's protocol. After EdU labeling, cells were fixed, permeabilized, and stained for EdU incorporation, followed by Hoechst counterstaining for nuclear visualization. Fluorescence images were acquired using identical imaging settings across groups.For analysis, nuclei were segmented in ImageJ using the Hoechst channel, and the mean EdU fluorescence intensity was quantified for each nucleus. A histogram of mean nuclear EdU intensity from the control group was generated and fitted with a bimodal Gaussian distribution. The intersection of the two Gaussian peaks was used as the threshold to distinguish EdU-negative and EdU-positive cells. Based on this threshold, the percentage of EdU-positive cells was calculated in each group.

Enzyme-linked Immunosorbent Assay (ELISA)
Collect LOVO cell supernatant, centrifuge at 1000g for 20 min at 2-8°C, and discard the precipitate. The amount of MUC2 in the cell supernatant was detected according to the instructions of MUC2 ELISA kit (Elabscience, E-EL-H0632c), and each experiment was repeated 3 times. Use Curve Expert software to plot the ELISA standard curve and calculate the concentration of target protein in the sample.

StcE Digestion Assay
Cells were treated with 5 µg/mL StcE (Cusabio, CSB-EP530572EOD) in medium for 2 h at 37°C.
Lysis was performed in buffer containing 50 mM Tris-Cl pH 8.0, 1% NP-40, 0.3% Triton X-100, 3% glycerol, and 150 mM NaCl on ice for 30 min[16]. Supernatants were collected after centrifugation at 25,000 g for 25 min at 4°C and analyzed by Western blot.

Chromatin Immunoprecipitation (ChIP) Assay
ChIP was conducted using the ChIP Assay Kit (Beyotime, P2078). LOVO cells were cultured to approximately 80% confluence and cross-linked with 1% formaldehyde for 10 minutes at 37°C to fix protein-DNA interactions. Cross-linking was quenched by adding glycine to a final concentration of 0.125 M and incubating at room temperature for 10 minutes. Cells were washed with cold PBS containing 1 mM PMSF and harvested by scraping. Cell pellets were lysed, and chromatin was sheared by sonication into 200–500 bp fragments using a probe sonicator. The lysates were pre-cleared and incubated overnight at 4°C with specific antibodies targeting transcription factors of interest (see Supplementary Table 2), followed by incubation with Protein A/G magnetic beads. After extensive washing, the chromatin complexes were eluted and cross-links reversed by incubation at 65°C overnight. Proteins were digested with proteinase K, and DNA was purified using a DNA extraction kit (Vazyme, DC301-01). Enrichment of target genomic regions was determined by quantitative PCR using gene-specific primers and DNA polymerase (Vazyme, P505-D1). PCR products were visualized by agarose gel electrophoresis or analyzed on a real-time PCR system. Primer sequences are listed in Supplementary Table 3.

Luciferase Reporter Assay

Promoter activity was analyzed using a dual-luciferase reporter system. Genomic DNA was extracted from LOVO cells using a genomic DNA extraction kit (Tiangen, DP304). Promoter regions encompassing 2 kb upstream and 100 bp downstream of the transcription start site were amplified by PCR using high-fidelity DNA polymerase and specific primers (Vazyme, P505-D1), and cloned into the pGL3-Basic luciferase reporter plasmid (Promega). To assess the role of transcription factor binding sites, site-directed mutagenesis was performed using a mutagenesis kit (Vazyme, C215-01) to introduce point mutations. The wild-type or mutant plasmids were co-transfected with the pRL-TK Renilla luciferase control plasmid into LOVO cells using JetPRIME (Polyplus). After 48 hours, cells were lysed and luciferase activities were measured using the Dual-Luciferase Reporter Assay System (UEBlandy, UE-F6075) on a luminometer. Firefly luciferase activity was normalized to Renilla luciferase to control for transfection efficiency.

Co-immunoprecipitation (Co-IP)

After transfection of pcDNA3.1-3xFlag-B3GNT6 plasmid in LOVO cells for 48 h, cells were lysed in cell lysis buffer for Western and IP (Beyotime, P0013) for 1 h. The lysate was collected, and the supernatant was obtained by centrifugation (12,000g, 25 min) at 4°C. The anti-Flag immunomagnetic beads (Selleck, B26101) were washed three times for 3 min each using the same lysis buffer on a DNA mixer. The beads were then incubated with 4 μg of IgG antibody (Proteintech, B900620) or 4 μg of B3GNT6 antibody (Cusabio, CSB-PA744392LA01HU) on the DNA mixer for 3 h. Subsequently, the cell protein lysate was added, and the mixture was incubated overnight at 4°C. The beads were washed seven times for 5 min each using lysis buffer on the DNA mixer. After discarding the supernatant, 20 μL of lysis buffer and 5 μL of Protein Loading Buffer (New Cell & Molecular Biotech, WB2001) were added, and the mixture was boiled for 10 minutes to denature the proteins. Western blotting was performed subsequently.

Mice Models

All animal experiments were conducted in accordance with the Regulations for the Administration of Affairs Concerning Experimental Animals of China. The study protocol was approved by the Institutional Animal Care and Use Committee of Central South University (Approval No. CSU-2022-0684). A total of 30 female C57BL/6J mice (6–8 weeks old, weighing 18–22 g) were purchased from the Department of Laboratory Animals, Central South University. The animals were specific-pathogen-free (SPF), nulliparous, and non-pregnant. All mice were housed in the Experimental Animal Center of Central South University under controlled conditions (23 ± 3°C, 55 ± 15% humidity) with a 12-hour light/dark cycle. Animals were maintained in individually ventilated cages with autoclaved bedding, and standard laboratory chow and water were provided ad libitum. Bedding was replaced every 2 days.

For in vivo dietary intervention, mice were randomly divided into three groups: control, rosiglitazone-treated, and GW9662-treated groups (n = 10 per group). The treatment groups were administered a standard diet containing rosiglitazone (10 mg/kg) or GW9662 (10 mg/kg) for two weeks. At the end of the treatment period, mice were euthanized by CO_2 asphyxiation. Intestinal tissues were collected and fixed for histological analysis. Immunohistochemistry was performed to detect expression levels of PPARγ, KLF4, B3GNT6, and MUC2. Lectin histochemistry was conducted using BSL-II to assess glycosylation profiles. MUC2 secretion levels in intestinal mucus were quantified using ELISA (Elabscience, E-EL-H0632c).

Xenograft Tumor Model

To generate stable B3GNT6-overexpressing CRC cells, the full-length human B3GNT6 coding sequence was cloned into a lentiviral expression vector. Lentiviral particles were produced and used to infect LOVO cells according to the manufacturer's instructions. After infection, cells were selected with the appropriate antibiotic to establish a stable B3GNT6-overexpressing cell line. Control cells were generated in parallel using the corresponding empty vector. To evaluate the effect of B3GNT6 on tumor growth in vivo, a subcutaneous xenograft model was established using LOVO cells stably overexpressing B3GNT6. Briefly, control LOVO cells and B3GNT6-overexpressing LOVO cells were harvested during the logarithmic growth phase, washed twice with(PBS, and resuspended in serum-free medium. A total of $5x10^6$ cells in 100 μL suspension were subcutaneously injected into the right flank of each female BALB/c nude mouse (4-6 weeks old). Mice were randomly assigned to the control group and the B3GNT6 overexpression group. At the experimental endpoint, mice were euthanized by CO_2 asphyxiation, and xenograft tumors were excised, photographed, and weighed. Tumor tissues were then fixed in 4% paraformaldehyde, embedded in paraffin, and sectioned for further histological analyses.Immunohistochemistry was performed to detect the expression levels of B3GNT6 and MUC2 in xenograft tissues. Lectin histochemistry was conducted using BSL-II to evaluate mucin-associated glycosylation levels. The staining results were semi-quantitatively analyzed as described above.

AOM/DSS Model

An azoxymethane (AOM)/dextran sulfate sodium (DSS)-induced colitis-associated colorectal cancer model was established to evaluate in vivo tumorigenesis. Following a one-week acclimatization period, female C57BL/6J mice (6–8 weeks old, 18–22 g) were randomly assigned to treatment and control groups. Mice in the experimental group were administered rosiglitazone (10 mg/kg) daily by oral gavage starting three days before AOM injection. On day 0, all mice received a single subcutaneous injection of AOM (12 mg/kg; Abiowell, AWH1007a, CAS: 25843-45-2). One week later, 3% DSS (Meilunbio, MB0842, CAS: 68528-80-3) was provided in the drinking water ad libitum for 7 days, followed by 2 weeks of regular drinking water. This 3-week cycle was repeated for a total of three cycles (65 days).

Six mice were randomly euthanized on the last day of each DSS cycle (days 23, 44, and 65 post-AOM). Mice were also euthanized if humane endpoints were reached, including ≥20–25% body weight loss, tumor volume exceeding 10% of body weight, tumor area >2000 mm^3, or tumor diameter >20 mm. After euthanasia, intestines were longitudinally opened and rinsed with PBS. Tumor number and diameter were recorded. Fecal samples were collected from the intestinal lumen for analysis. Occult blood was assessed using a Fecal Occult Blood Kit (Leagene Biotechnology), and disease activity index (DAI) scores were calculated based on body weight loss, stool consistency, and presence of blood.

Histological Scoring

Histological evaluation of colonic inflammation and epithelial damage was performed on paraffin-embedded sections stained with hematoxylin and eosin (HE) using the Hematoxylin-Eosin Staining Kit (Solarbio, G1120), following the manufacturer's instructions. Two experienced and blinded pathologists independently assessed the stained sections using the established colon pathology scoring criteria described by Horino et al[17] , with details summarized in Supplementary Table S1. Scoring parameters included the severity and extent of inflammatory cell infiltration, epithelial crypt loss, and mucosal architecture disruption.

16S Sequencing Analysis

16S rRNA gene sequencing was performed to assess the composition and diversity of the gut microbiota. Cecal contents were collected from euthanized mice under sterile conditions, and total microbial DNA was extracted using the QIAamp DNA Stool Mini Kit (QIAGEN, US) according to the manufacturer's instructions. The V3–V4 hypervariable region of the bacterial 16S rRNA gene was amplified by PCR using universal primers: 341F (5'-CCTACGGGNGGCWGCAG-3') and 805R (5'-GACTACHVGGGTATTCTAATCC-3'). PCR products were evaluated by electrophoresis on a 2% agarose gel, and target fragments were purified using the AxyPrep PCR Clean-up Kit (Axygen, US). Purified amplicons were quantified using the Quant-iT PicoGreen dsDNA Assay Kit (Invitrogen, US) and analyzed on the Qubit Fluorometer (Thermo Fisher Scientific). Sequencing libraries were constructed and paired-end sequencing was conducted by Lc-Bio Technologies Co., Ltd. (Hangzhou, China). The resulting raw data were demultiplexed and processed using QIIME2 (v2021.11) for quality filtering, feature table construction, and taxonomic classification. Alpha diversity and beta diversity analyses were performed, and visualizations were generated using the R package (v3.5.2).

Statistics

All quantitative data are presented as mean ± standard error of the mean (SEM), unless otherwise stated, and derived from at least three independent biological replicates. Statistical analyses were conducted using GraphPad Prism (version 7.0; GraphPad Software, USA). A paired two-tailed Student's t-test was used for experiments comparing matched normal and tumor tissues (e.g., IHC, Alcian Blue staining, and Western blot analyses in Figures 1, 2, 3, and 4). For comparisons among three or more groups, one-way ANOVA followed by Tukey's post-hoc test was applied, including in most cell-based assays (e.g., luciferase assays, ELISA, qPCR, CCK-8, colony formation, and ChIP-qPCR) and dose-response experiments (Figures 2–6 and Supplementary Figures 1–6). Microbiome data analyses (Figure 6) including alpha diversity (rarefaction curves), beta diversity (PCoA), and functional gene heatmaps (KEGG, CAZy) were performed using embedded statistical methods within standard metagenomics pipelines as described in the figure legend. The specific statistical test used for each dataset is stated in the corresponding figure legend. Significance was defined as $p < 0.05$. Significance levels are denoted as: $p < 0.05$ (*), $p < 0.01$ (**), and $p < 0.001$ (***).
