# Supplementary material for: Restoring O-glycosylation and expression of MUC2 limits progression of colorectal cancer
Source: Cell Death Dis. 2026 Jun 21;17(1):768. doi: 10.1038/s41419-026-08981-x (PMC13530250; doi:10.1038/s41419-026-08981-x)

Original Western Blot Data

Original Western blots

Figure1.E

MUC2

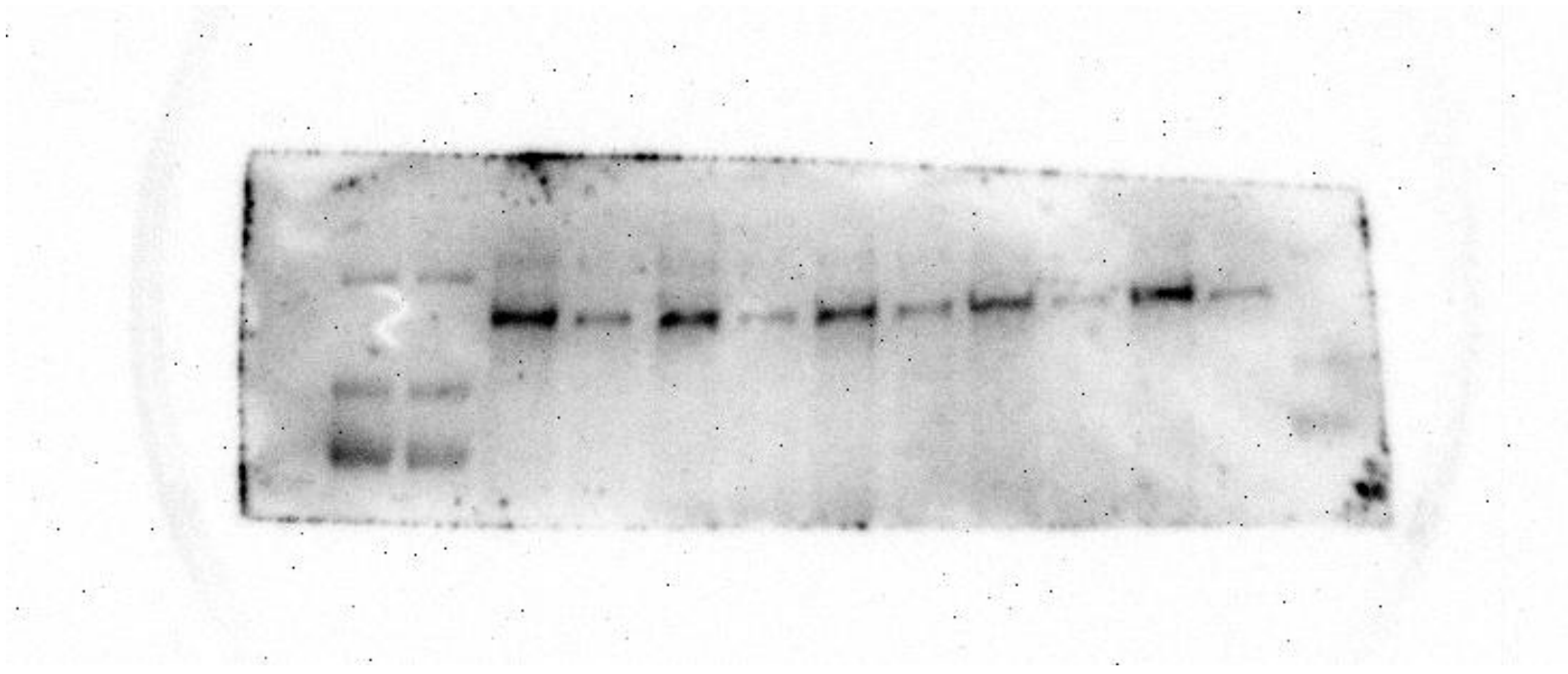

GAPDH

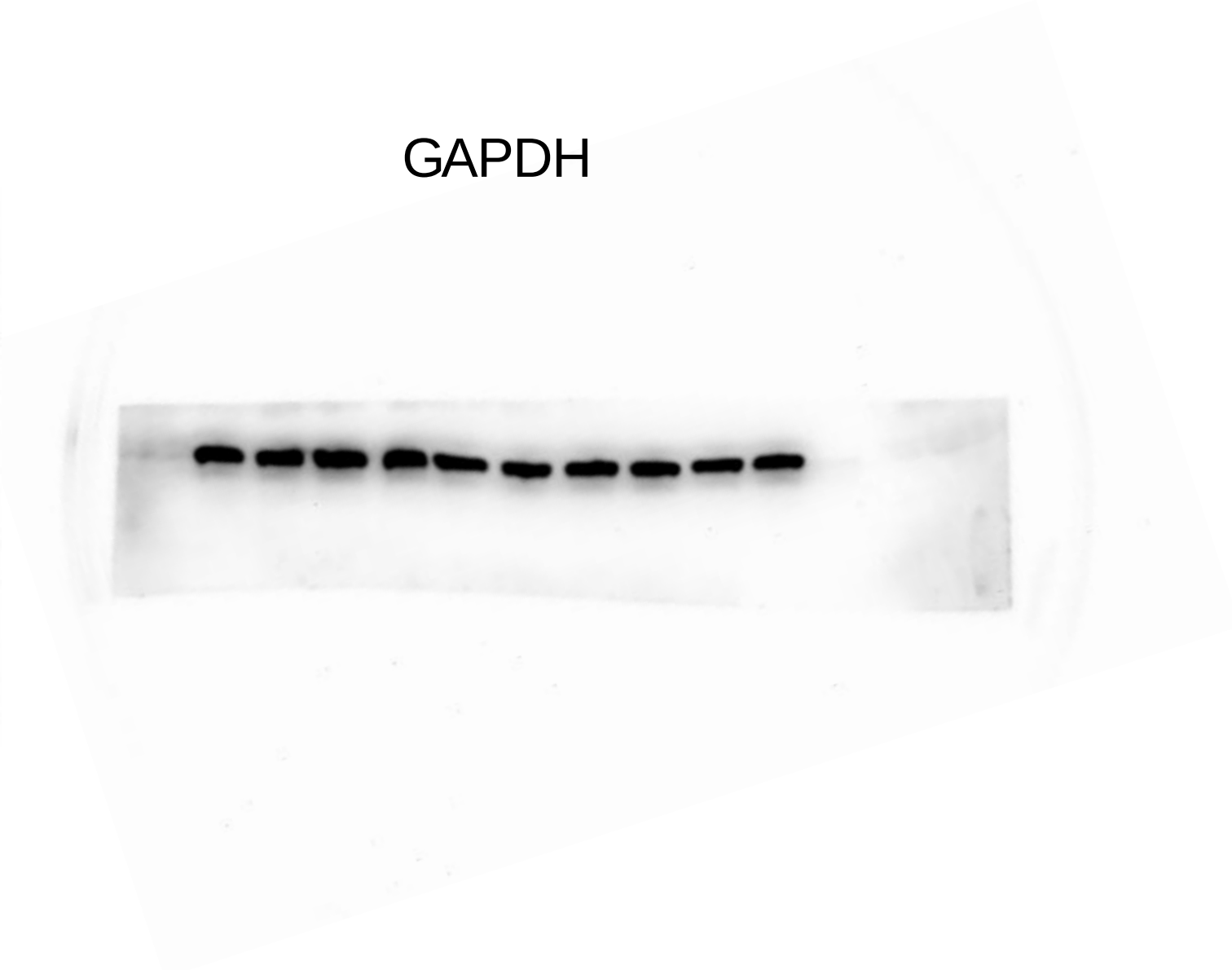

Figure2.E

B3GNT6

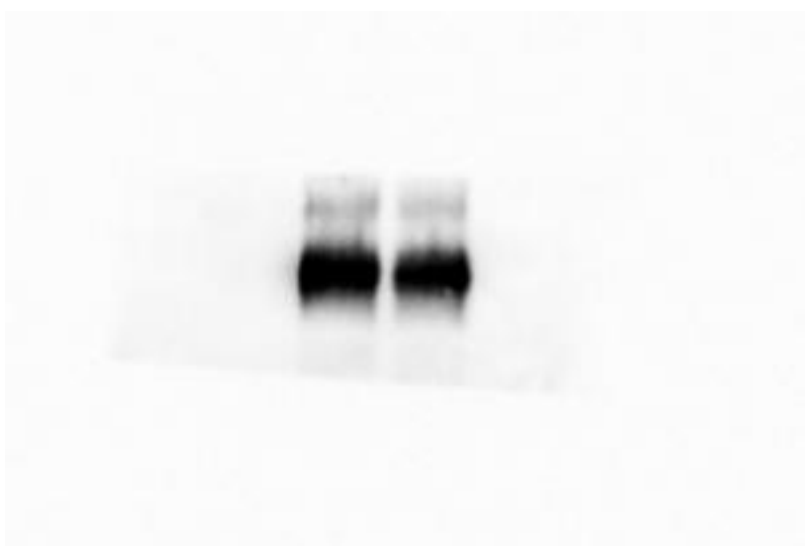

MUC2

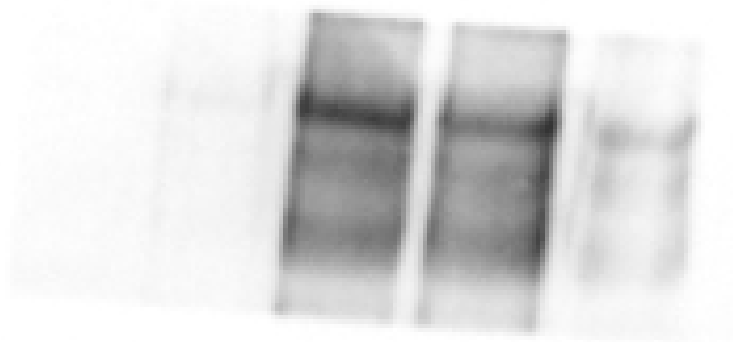

Figure2.F

Input-B3GNT6

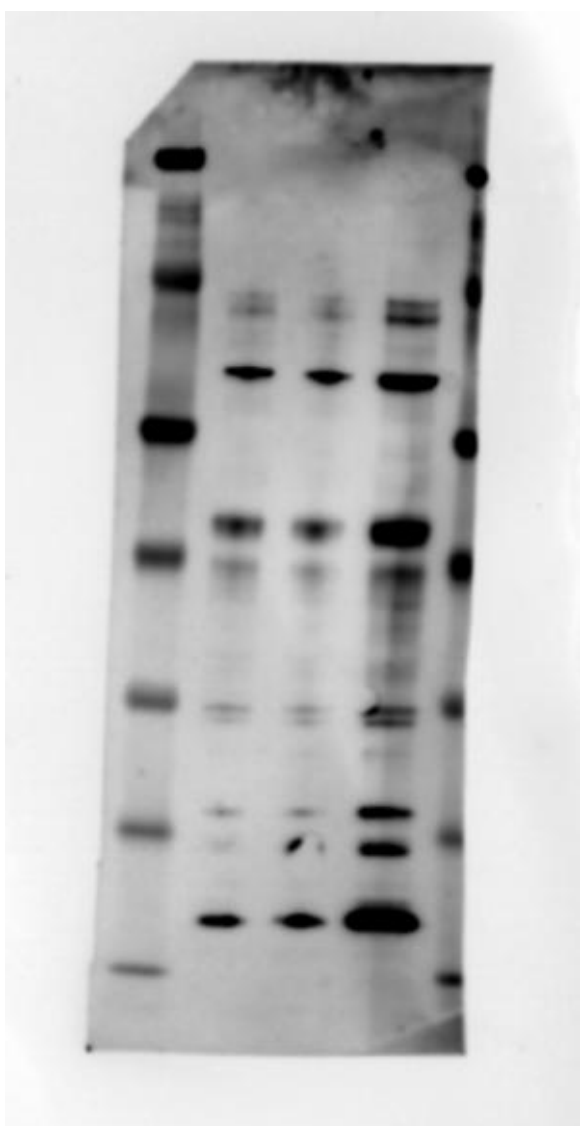

Input-MUC2

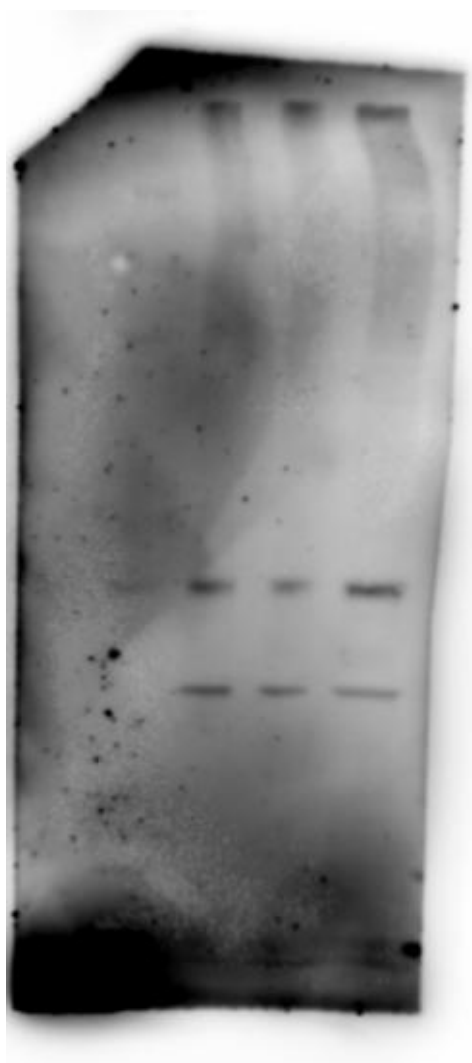

Input-GAPDH

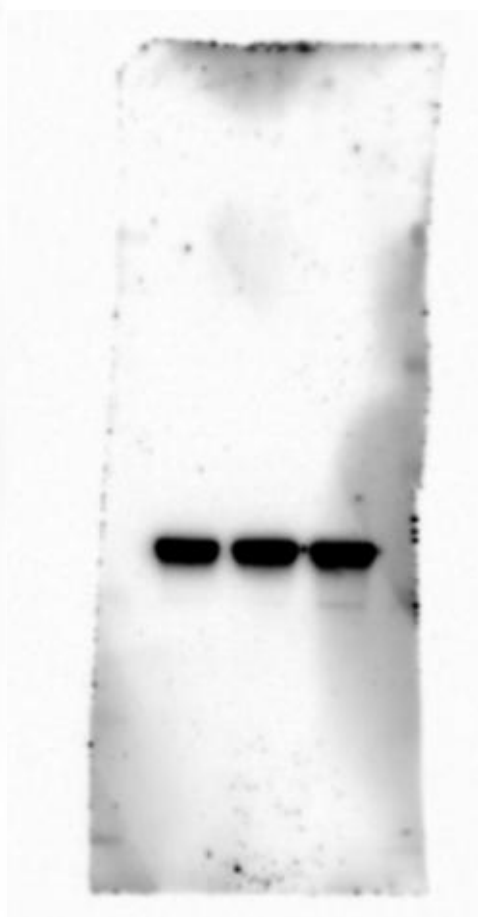

IP-BSL2

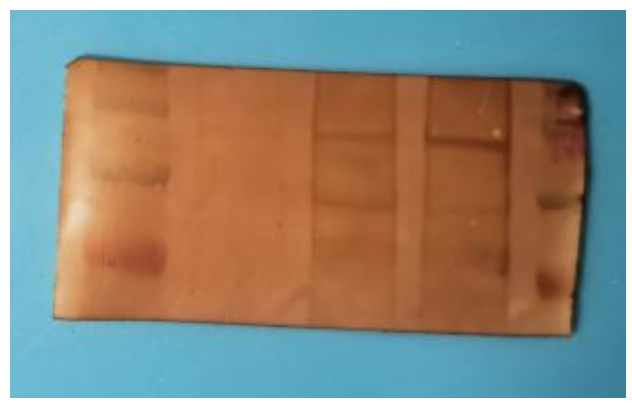

IP-MUC2

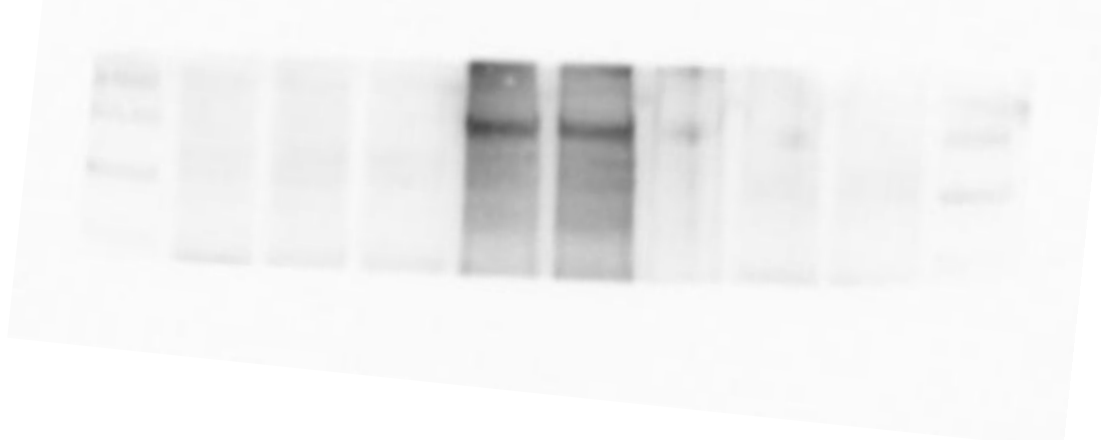

Figure2.H

Vector MUC2

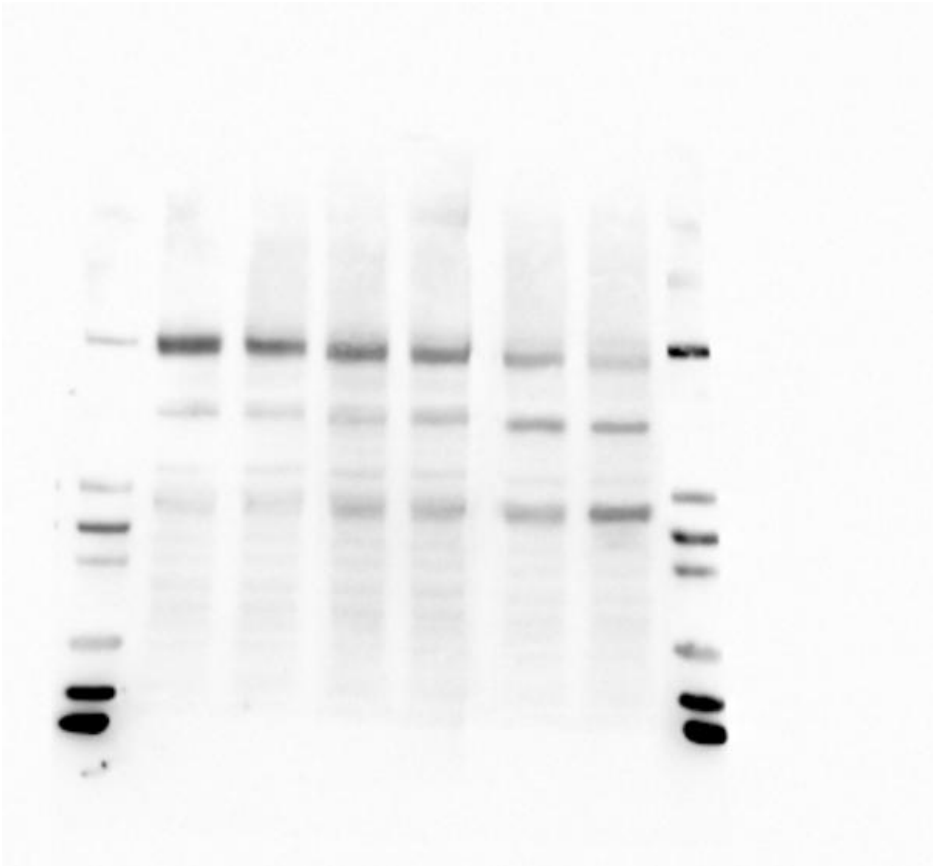

B3GNT6-OE MUC2

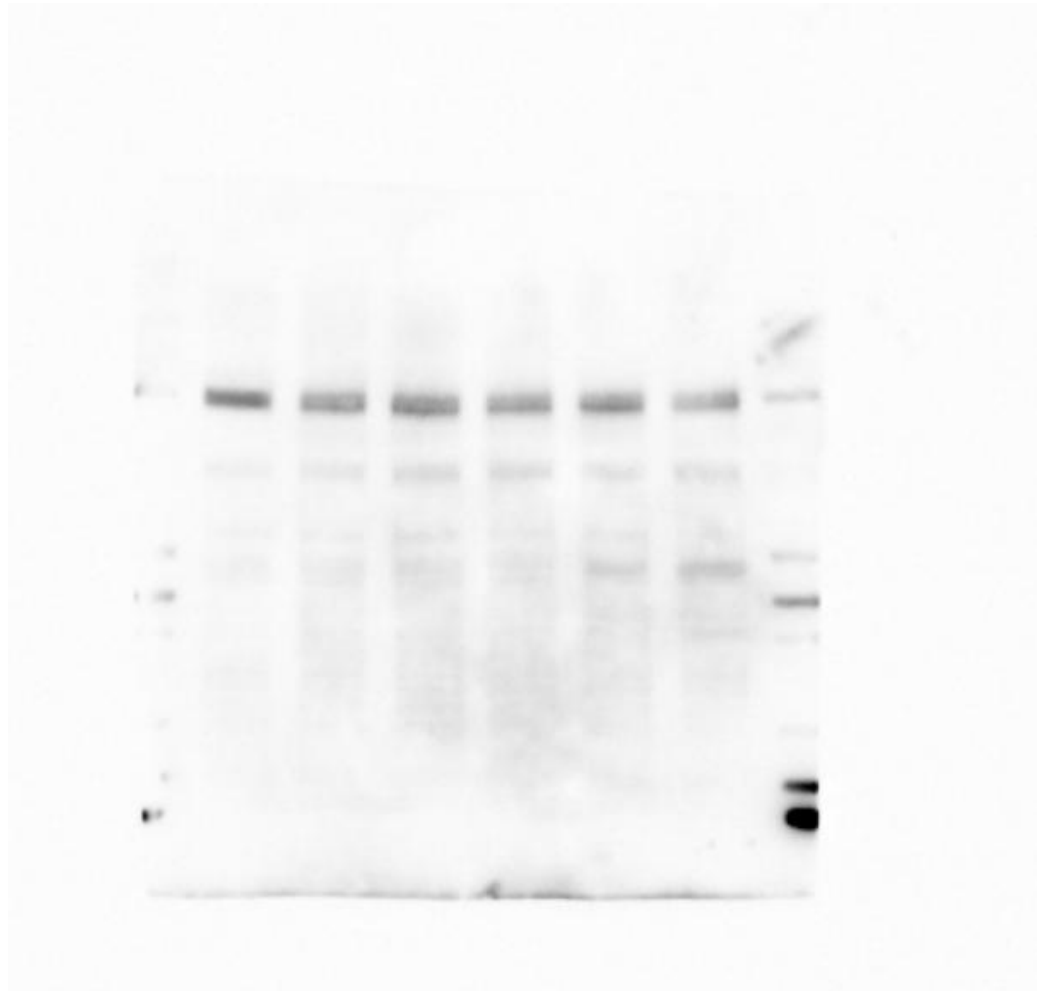

Vector GAPDH

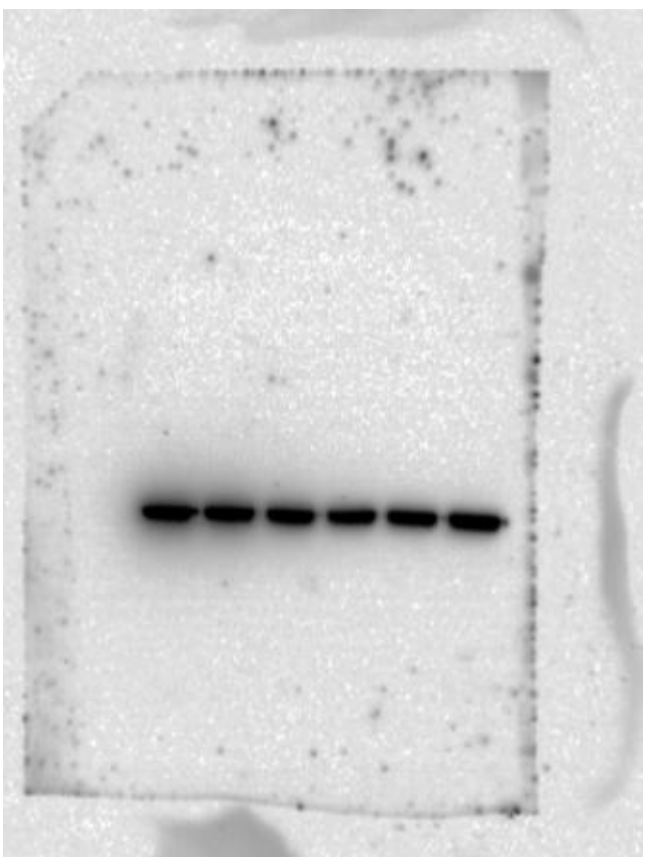

B3GNT6-OE GAPDH

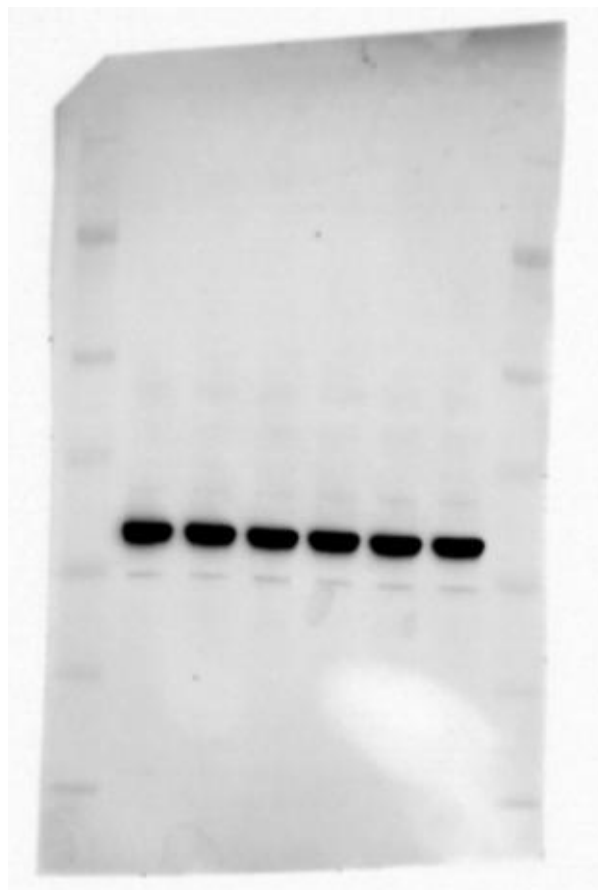

Figure4.C

KLF4

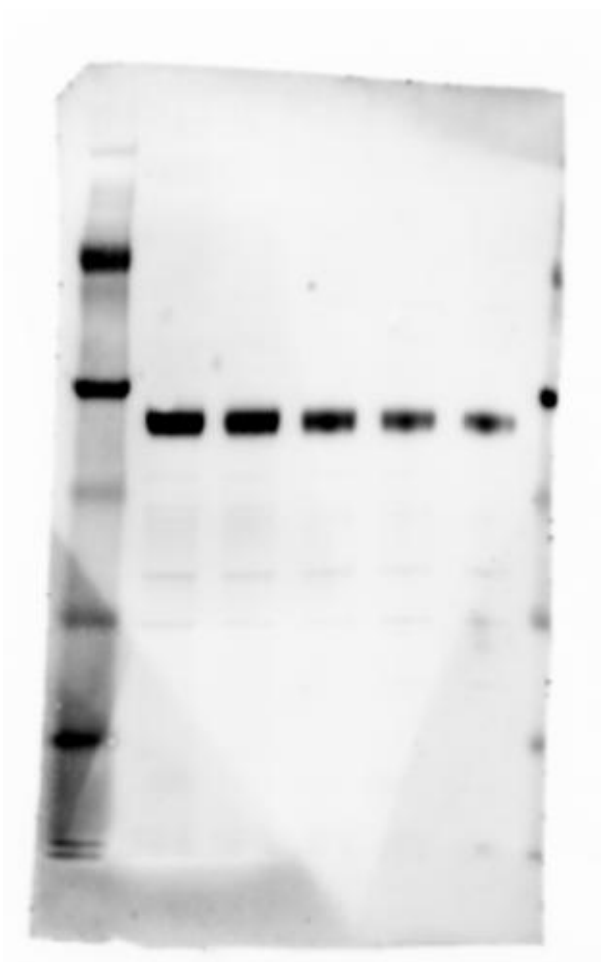

MUC2

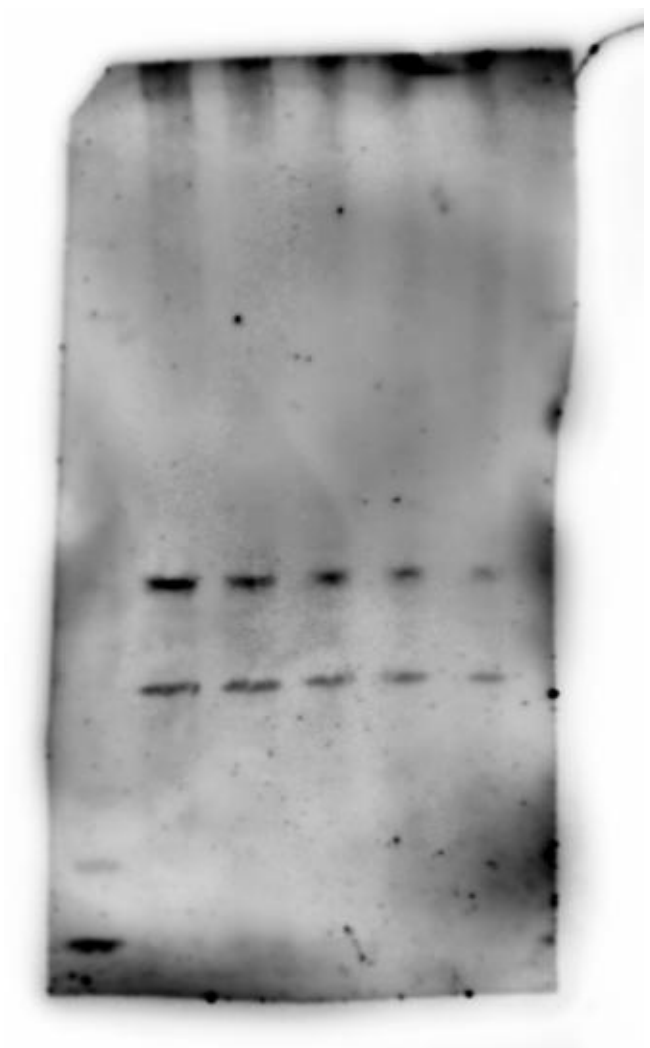

GAPDH

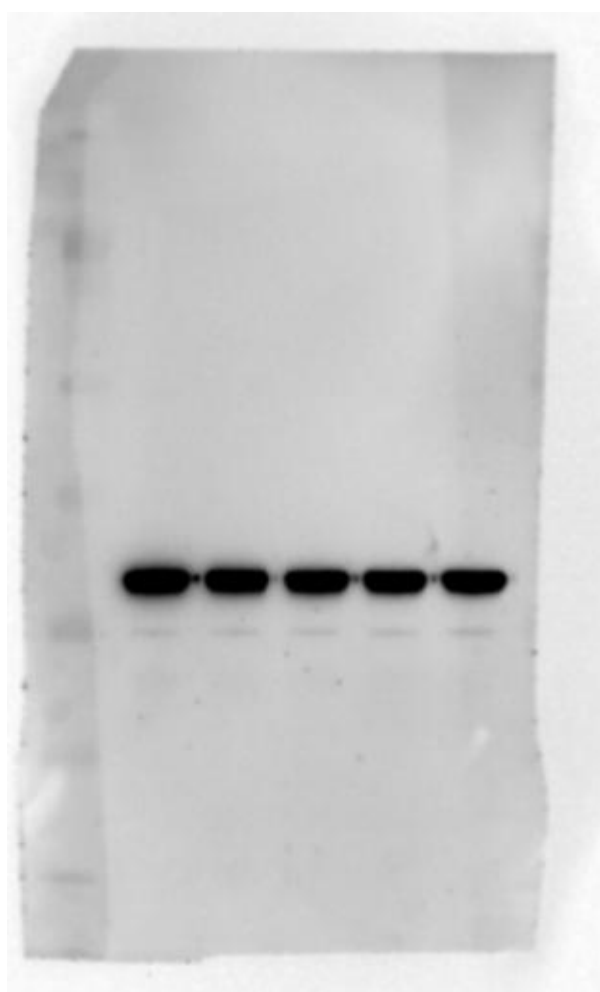

Figure5.C

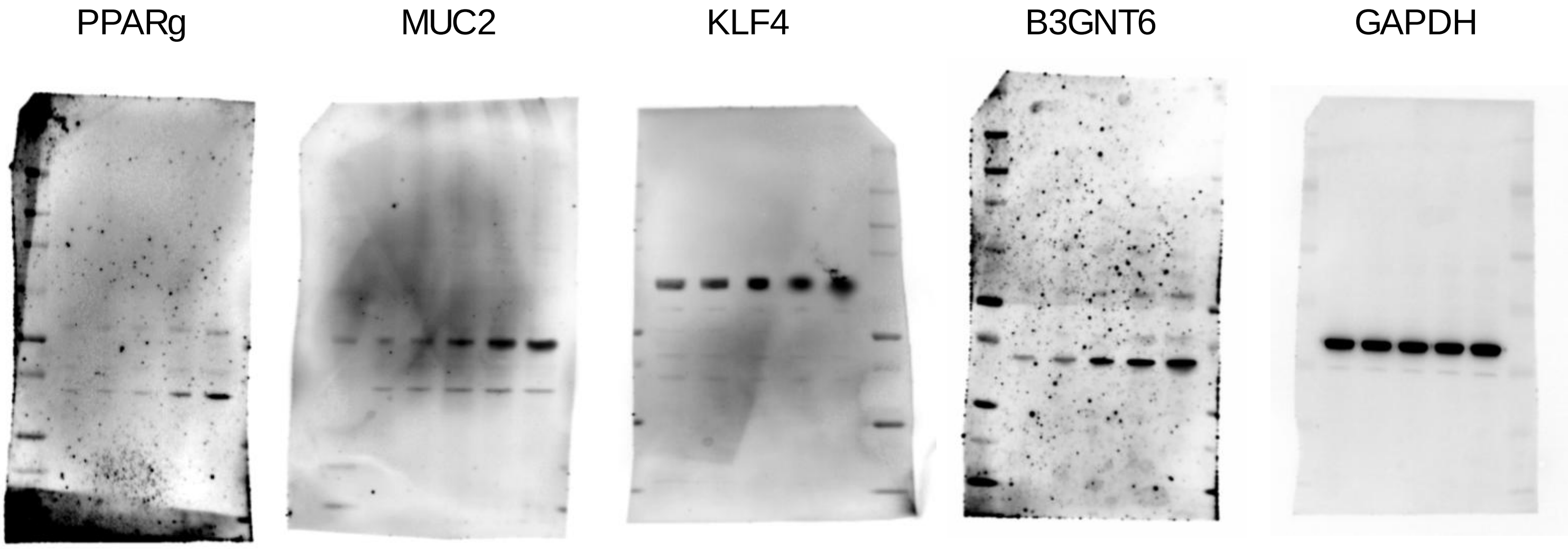

Figure5.D

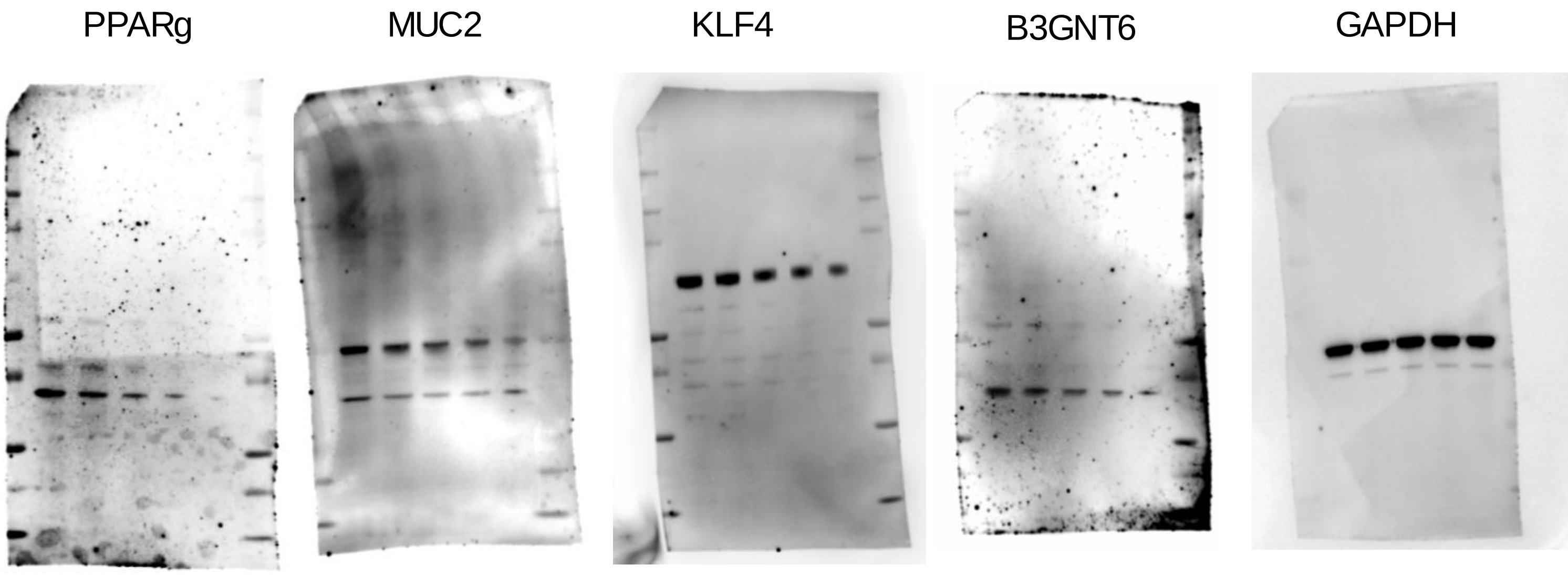

Figure5.H

PPARg

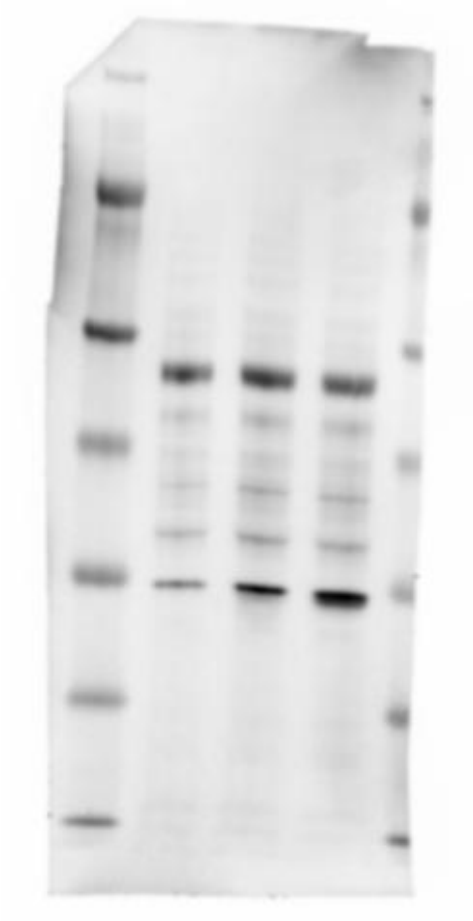

KLF4

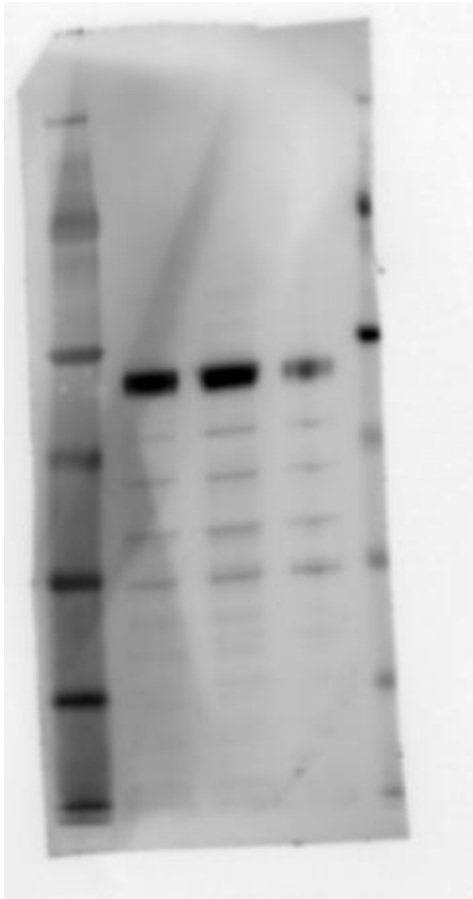

MUC2

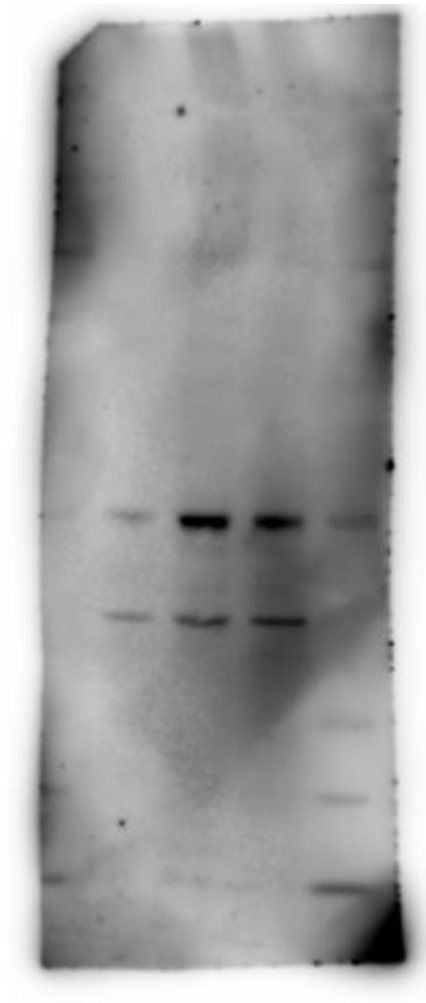

GAPDH

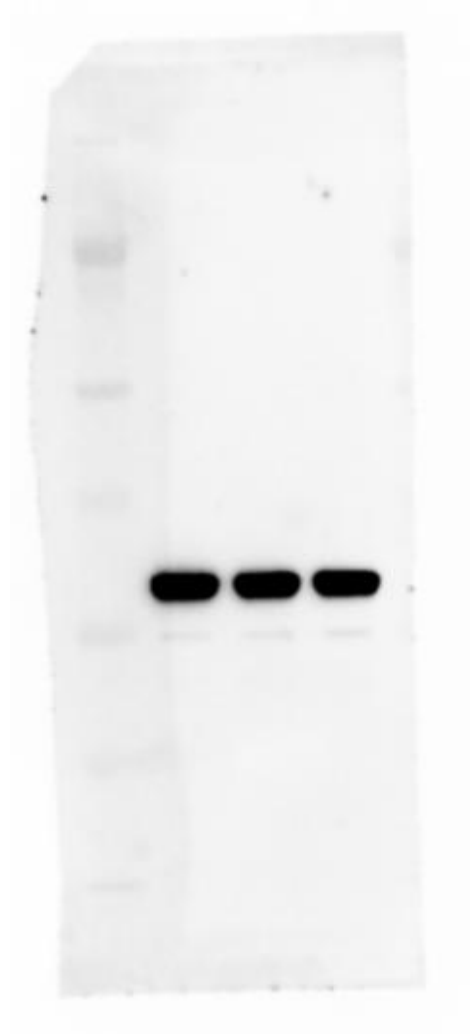

Figure5.I

PPARg

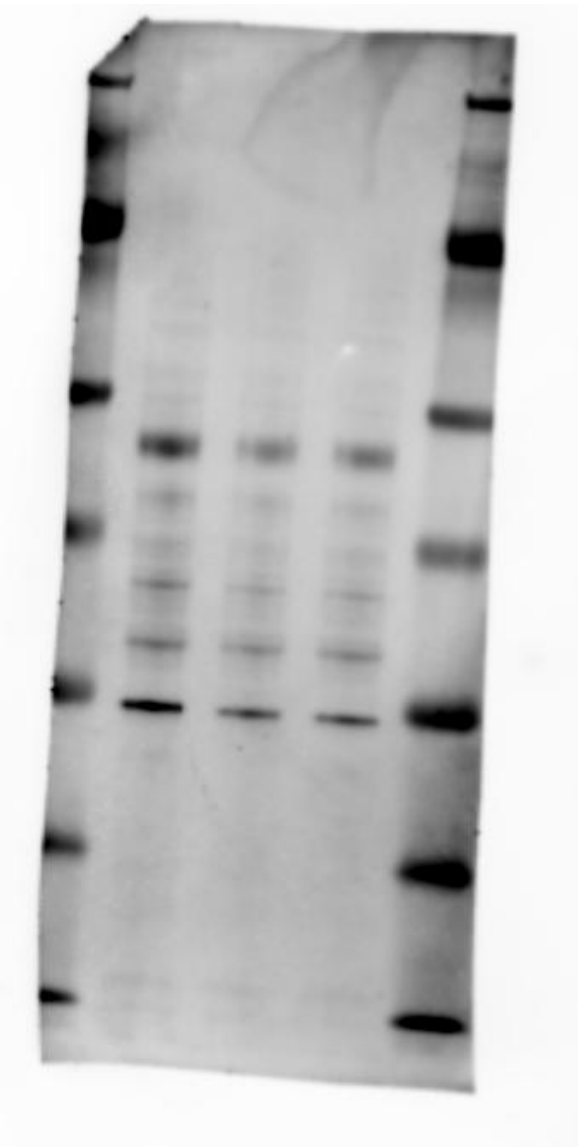

KLF4

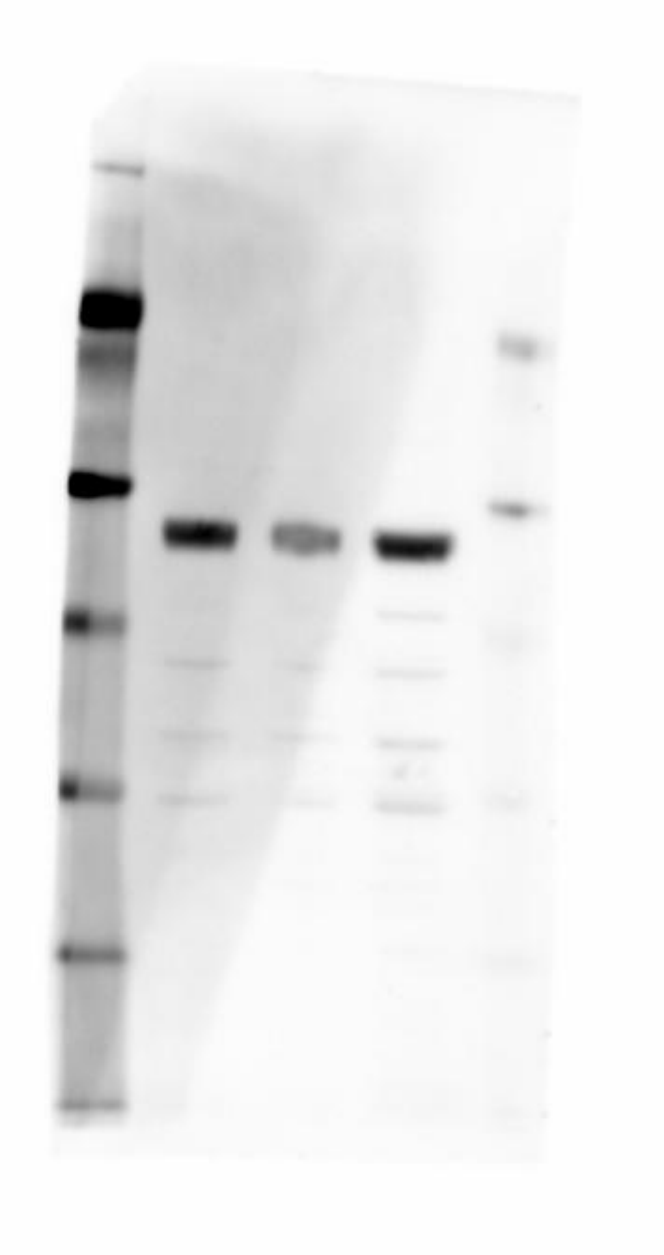

MUC2

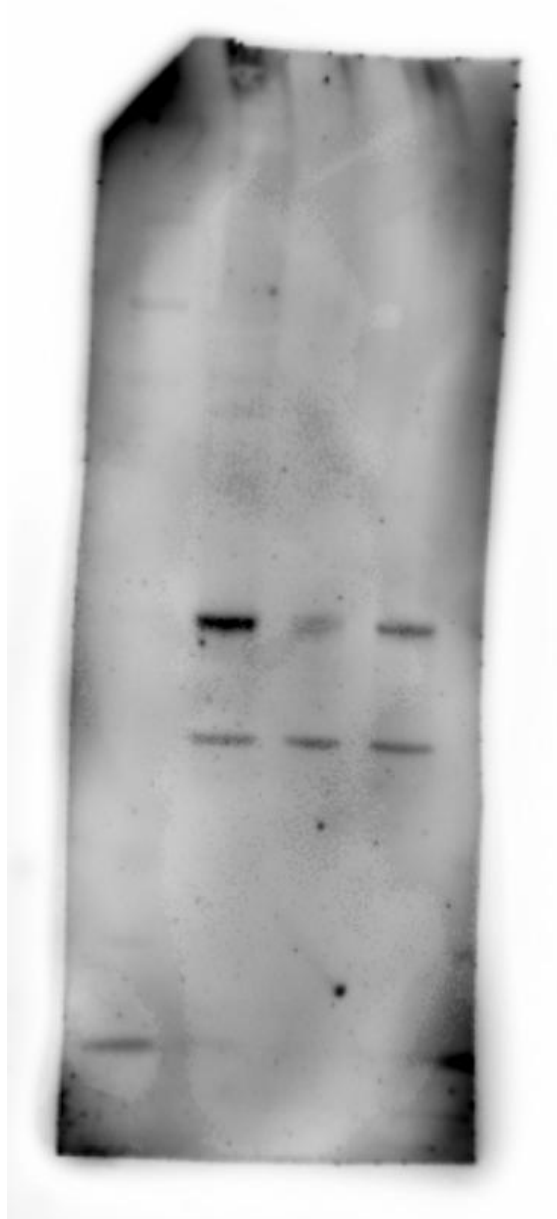

GAPDH

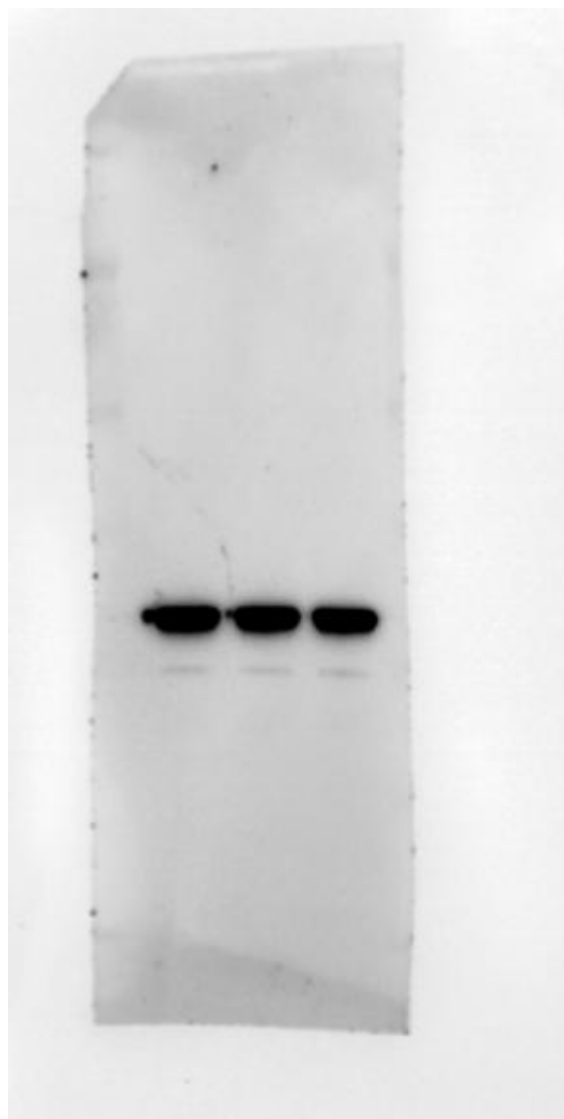

Supplementary Figure3.B

B3GNT6

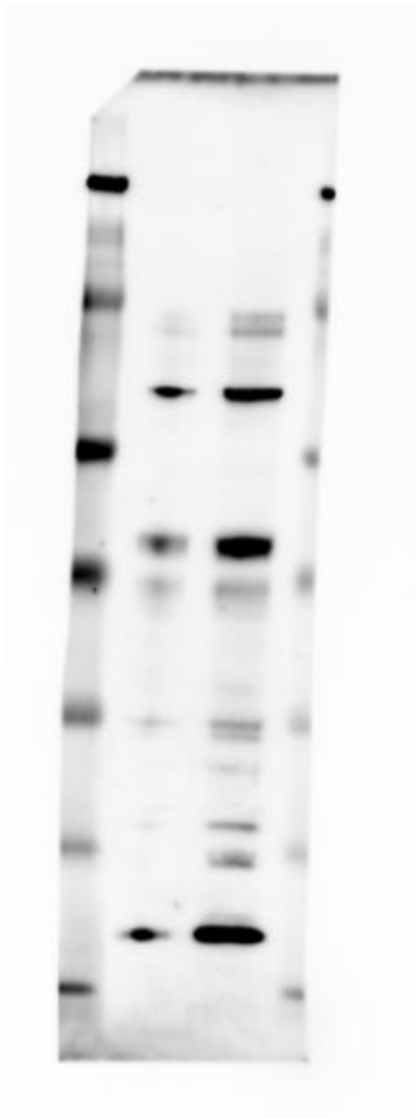

MUC2

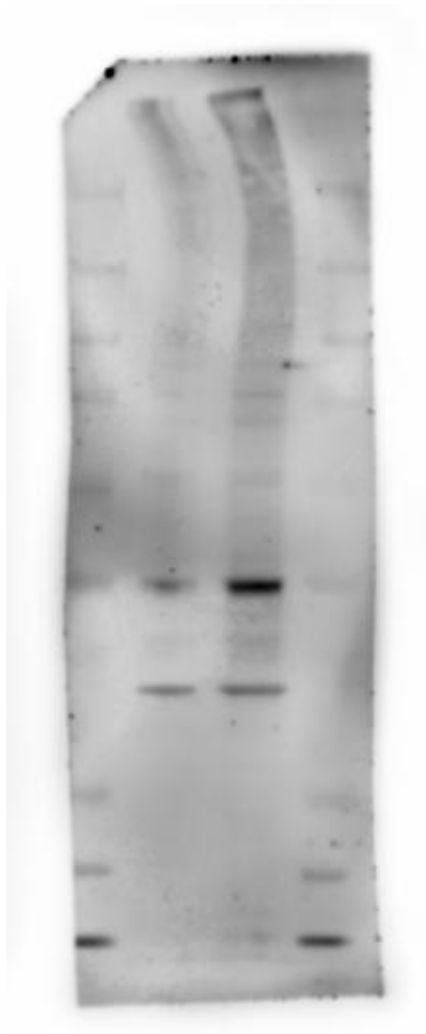

GAPDH

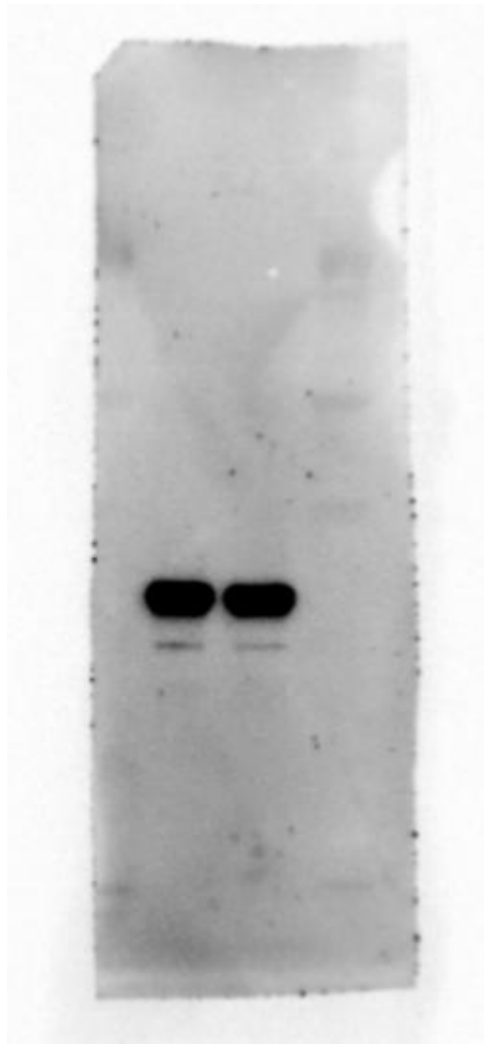

Supplementary Figure6.G

KLF4

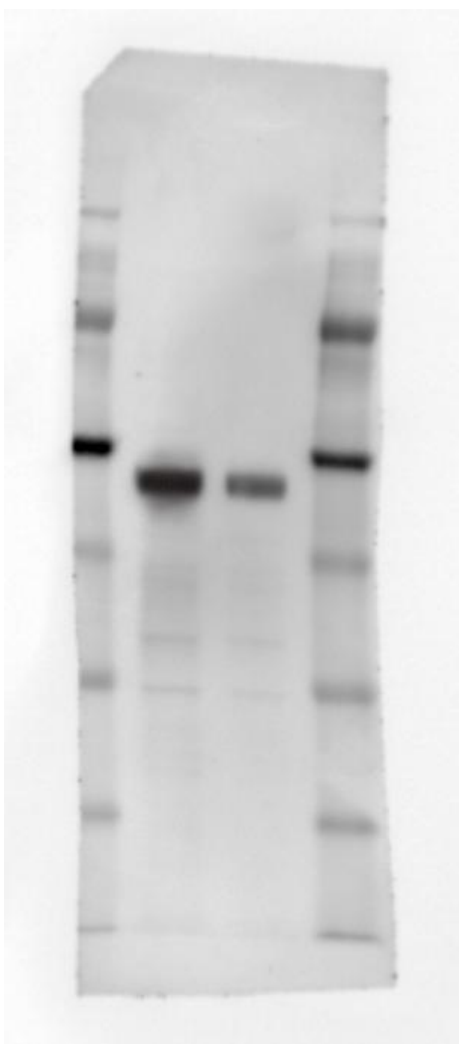

MUC2

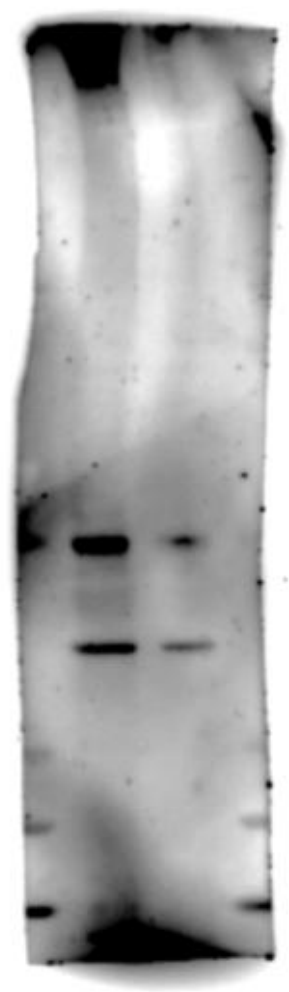

GAPDH

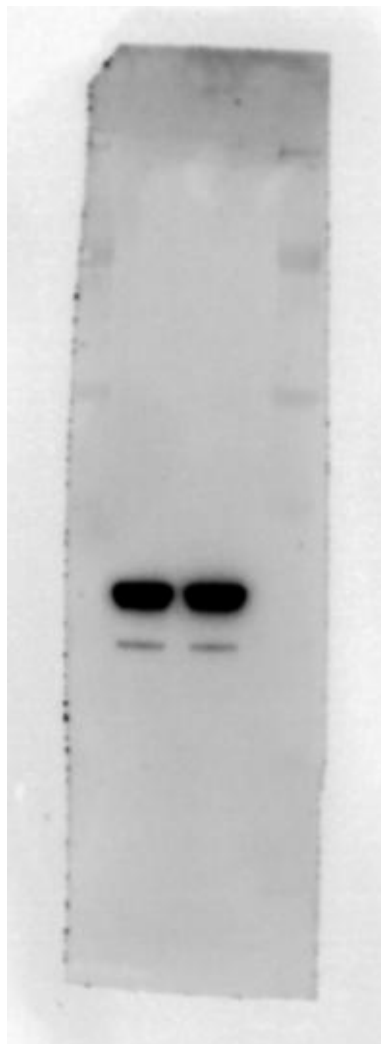

Supplementary Figure6.I

KLF4

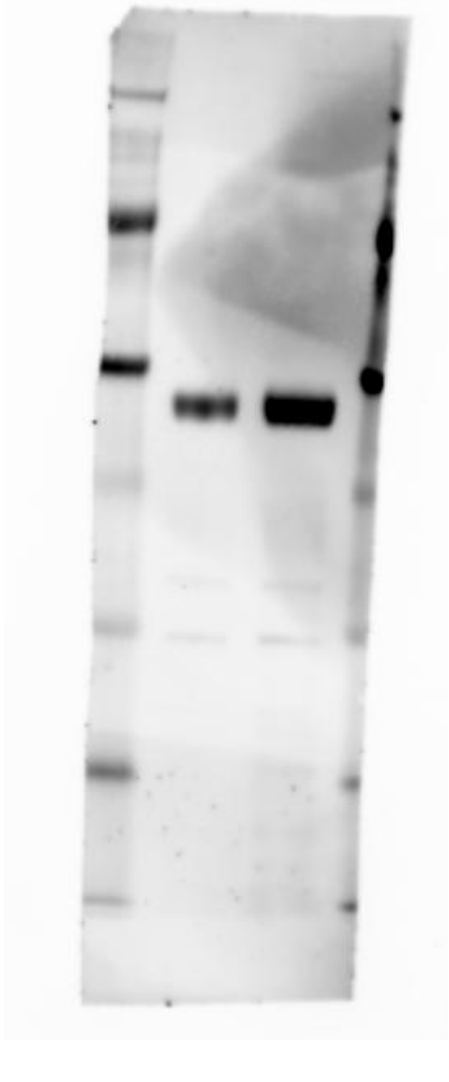

MUC2

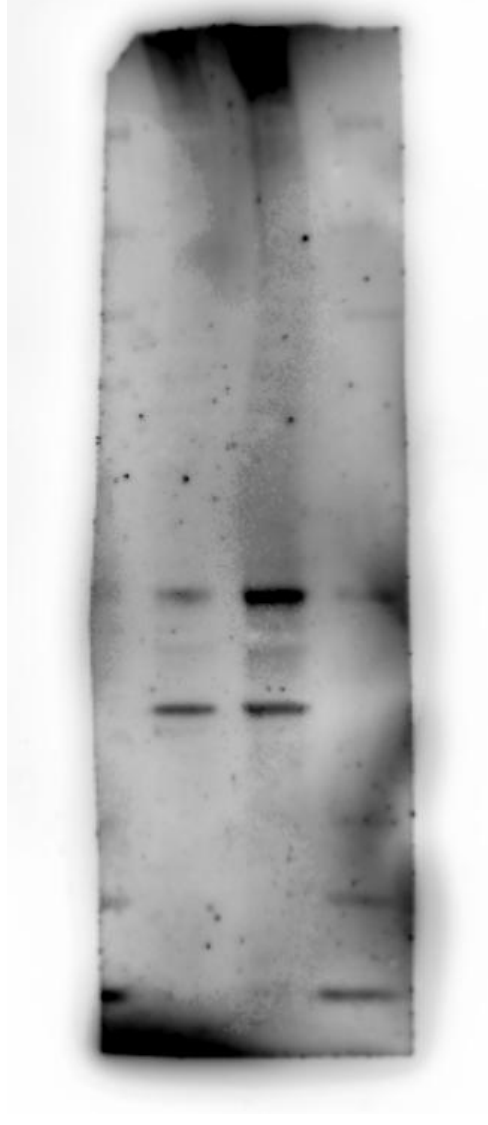

GAPDH

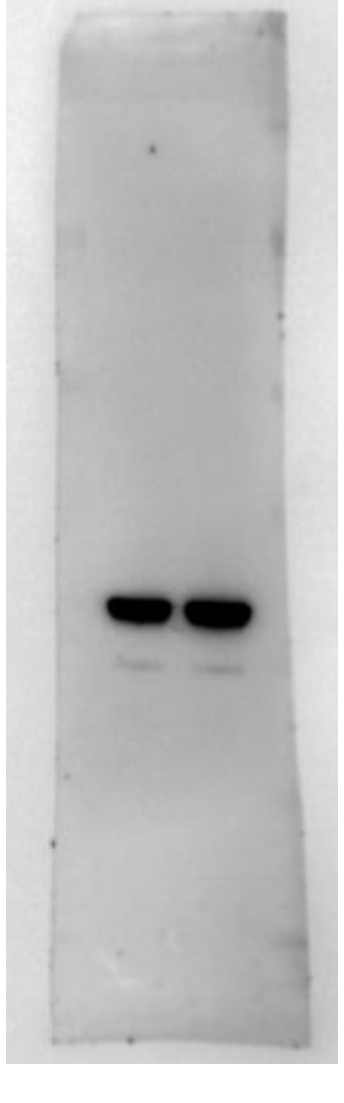

Supplementary Figure6.L

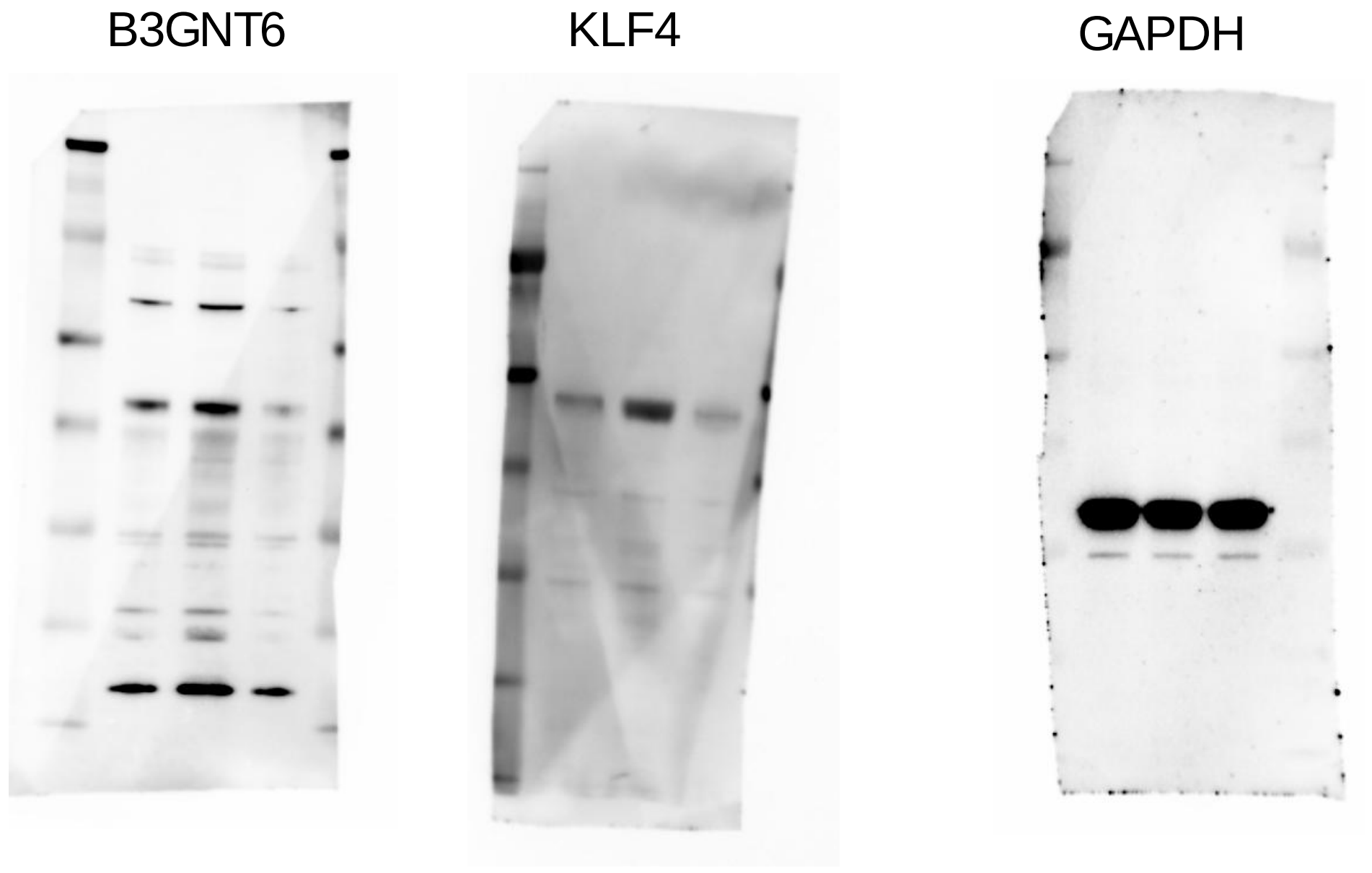

Supplement: Supplementary file 2 [file 41419_2026_8981_MOESM2_ESM.pdf]
